# Ecological Diversity in South American Mammals: Their Geographical Distribution Shows Variable Associations with Phylogenetic Diversity and Does Not Follow the Latitudinal Richness Gradient

**DOI:** 10.1371/journal.pone.0128264

**Published:** 2015-06-08

**Authors:** Paula Nilda Fergnani, Adriana Ruggiero

**Affiliations:** Laboratorio Ecotono, Centro Regional Universitario Bariloche-Universidad Nacional del Comahue, INIBIOMA-CONICET, San Carlos de Bariloche, Argentina; Smithsonian Institution, UNITED STATES

## Abstract

The extent to which the latitudinal gradient in species richness may be paralleled by a similar gradient of increasing functional or phylogenetic diversity is a matter of controversy. We evaluated whether taxonomic richness (TR) is informative in terms of ecological diversity (ED, an approximation to functional diversity) and phylogenetic diversity (AvPD) using data on 531 mammal species representing South American old autochthonous (marsupials, xenarthrans), mid-Cenozoic immigrants (hystricognaths, primates) and newcomers (carnivorans, artiodactyls). If closely related species are ecologically more similar than distantly related species, AvPD will be a strong predictor of ED; however, lower ED than predicted from AvPD may be due to species retaining most of their ancestral characters, suggesting niche conservatism. This pattern could occur in tropical rainforests for taxa of tropical affinity (old autochthonous and mid-Cenozoic immigrants) and in open and arid habitats for newcomers. In contrast, higher ED than expected from AvPD could occur, possibly in association with niche evolution, in arid and open habitats for taxa of tropical affinity and in forested habitats for newcomers. We found that TR was a poor predictor of ED and AvPD. After controlling for TR, there was considerable variability in the extent to which AvPD accounted for ED. Taxa of tropical affinity did not support the prediction of ED deficit within tropical rainforests, rather, they showed a mosaic of regions with an excess of ED interspersed with zones of ED deficit within the tropics; newcomers showed ED deficit in arid and open regions. Some taxa of tropical affinity showed excess of ED in tropical desert areas (hystricognaths) or temperate semideserts (xenarthrans); newcomers showed excess of ED at cold-temperate latitudes in the Northern Hemisphere. This result suggests that extreme climatic conditions at both temperate and tropical latitudes may have promoted niche evolution in mammals.

## Introduction

During the past decade, interest in the latitudinal diversity gradient has moved away from the exclusive elucidation of species richness patterns to a more integrative consideration of phylogenetic and functional differences among species. Functional and evolutionary components of diversity offer complementary information about how species differences influence patterns of community assembly and species coexistence [1, 2]. Until recently, however, much of the attention was focused on the establishment of formal definitions and measurements of different components of diversity (e.g., [3–5]). The initial emphasis on methodological issues and inherent difficulties in trying to define and quantify functional and phylogenetic diversity independently of species richness [2, 6–8] have somewhat complicated the assessment of conclusive associations between functional and historical components of species diversity [9, 10]. Thus, a debate is still pending on the issue of how different components of diversity are associated in different taxonomic groups across different regions of the world.

The latitudinal gradient in species richness may be paralleled by a similar gradient of increasing functional diversity towards the tropics if tropical species occupy more specialized niches than temperate species [11, 12]. On the other hand, patterns in phylogenetic diversity may parallel patterns in functional diversity, since more closely related species tend to be more similar in their ecology than more distantly related species (e.g., [13–15]). Although in theory a positive association between richness, functional and phylogenetic diversity is justified, there is nonetheless increasing evidence that suggests that variation in species richness cannot be considered a proxy for the variation in functional or phylogenetic diversity (e.g., [16–18]). For instance, the association between species richness and phylogenetic diversity could be weaker in the presence of recent speciation events or assembly processes that select for clades of closely related species [19], and phylogenetic diversity has also been found to vary independently of functional diversity (e.g., [2, 9]).

At present, two hypotheses have been proposed to account for geographical variability in the association between functional and phylogenetic diversity. The tropical niche conservatism hypothesis [13, 20, 21] posits that if tropical species retained most of their ancestral characters, this would lead to lower functional diversity than predicted from phylogenetic diversity at tropical latitudes [1]. In contrast, the niche evolution hypothesis predicts higher functional diversity than predicted from phylogenetic diversity whenever circumstances promote the appearance of new ecological niches, or when competition between ecologically similar species is strong [1, 10, 22, 23].

It has been suggested that tropical niche conservatism is important for mammal richness gradients [24]; however, analysis of phylogenetic structure suggested that high mammal richness within the tropics is attributable to many species from both a few basal groups and many derived groups arising in the Miocene and Pliocene [25]. This phylogenetic structure, along with the greater time and area available for speciation in the tropics [13], could account for the high mammal species richness, and high phylogenetic and ecological diversity within the tropics. Phylogenetic niche conservatism has also been invoked to play a key role in promoting vicariant isolation and speciation in landscapes where favourable habitats alternate with unfavourable habitats (e.g., montane regions) [13].

On the other hand, niche evolution enables the invasion of new habitats and climatic regimes that had previously limited the distribution of a clade, thus reducing competition and creating additional opportunities for speciation [26, 27]. Constrained evolution of tolerance of terrestrial organisms to high temperature could have resulted in niche evolution being more frequent at high latitudes [28]. Also, mammal species that have shifted their climatic constraints (with respect to their ancestors) are aggregated in northern North America [29]. On large temporal scales, during the Cenozoic arid climates and open habitats clearly arose more recently than environmental conditions similar to those of present-day tropical forests. The cooling and drying that promoted the expansion of extensive open habitats by Late Miocene may have triggered niche evolution at extra- tropical latitudes (mammals: [1], pitviper snakes: [30]), with the appearance and fixation of morphological and ecological specializations to open and arid habitats in some mammal groups (e.g., hystricognaths: [31] and references therein).

In this study we evaluated the relationships between taxonomic richness (TR), ecological diversity (ED) and phylogenetic diversity (AvPD) in mammal taxa with different histories of immigration in South America, and for which ecological data at species level were available. We recognized autochthonous xenarthrans and marsupials as living representatives of the oldest mammal lineages in South America, dating back to 65 mya [32, 33]. Mid-Cenozoic immigrants from Africa are represented by hystricognath rodents (whose earliest records in South America date back to about 41 mya [34]) and platyrrhine primates (ca. 29 mya [35]). “Newcomers” from North America are represented by artiodactyls and carnivorans that participated in the Great American Biotic Interchange (GABI) that began about 3.8 mya [36]. Throughout the present study we assumed that autochthonous taxa and mid-Cenozoic immigrants were taxa of tropical affinity, i.e. they faced environmental conditions similar to present-day tropical forests at the time of their arrival in South America; in contrast, the newcomers from North America were taxa with savanna-like ecologies that entered South America during glacial episodes and general climatic cooling [37].

We evaluated regional variability in the association between evolutionary and functional components of mammalian species diversity in order to address the extent to which ED and AvPD are positively associated, and to identify which regions show higher or lower ED than predicted by AvPD. We predicted three patterns of association between ED and AvPD, as follows:

1. Spatial patterns in phylogenetic diversity will parallel patterns in functional diversity if more closely related species are more similar in their ecology than less closely related species, e.g., [13–15]. We evaluated whether ED and AvPD are positively associated, showing congruent spatial patterns of variation. We tested for the generality of these associations for different mammal groups on a continental scale; however, as phylogenetic diversity has also been found to vary independently of functional diversity (e.g., [2, 9]), we also examined whether there was regional variability in the predictive ability of AvPD to account for ED, while controlling for TR.
2. If closely related species are ecologically more similar than expected based on their phylogenetic relationships, this will result in lower ED than predicted by AvPD [15]. Several factors (e.g. phylogenetic niche conservatism, relaxed competition, environmental filtering) may constrain divergence among closely related species [1]. If niche conservatism was predominant in South American mammals, this would predict that taxa of tropical affinity (marsupials, xenarthrans, hystricognaths and primates) would show lower ED than predicted from AvPD at tropical latitudes, mainly in tropical humid forests. In contrast, taxa that immigrated into South America through savanna-like environments (carnivorans and artiodactyls) would show lower ED than predicted from AvPD in open and arid habitats.
3. If closely related species are ecologically more dissimilar than expected based on their phylogenetic relationships, this will result in higher ED than predicted by AvPD. Several factors, including environmental limitation, competition, high environmental heterogeneity and niche evolution may account for the occurrence of an excess of ED [1, 13].

In the palaeoclimatic history of the Cenozoic in America, arid climates and open habitats arose more recently than environmental conditions similar to those of present-day tropical forests [38]. If the appearance of arid and open habitats was associated with niche evolution in South American mammals (e.g., hystricognaths [31]), we would predict a higher level of ED than predicted from AvPD in arid and open habitats for mammal taxa of tropical affinity, i.e. old autochthonous and mid-Cenozoic immigrants that faced environmental conditions similar to present-day tropical forests at the time of their first records in South America. Newcomers that entered South America through savanna-like environments could show an excess of ED in forest habitats.

## Methods

### Choice of taxa

We compiled data on the geographical distribution, functional attributes and phylogeny of 531 extant mammal species inhabiting North and South America, including marsupials (number of species, N = 78), xenarthrans (N = 29), artiodactyls (N = 29), carnivorans (N = 76), hystricognaths (N = 191) and primates (N = 128). The taxonomic list and the geographical distribution of species were taken from NatureServe [39], excluding exotic species and island endemic species. We also excluded two monotypic hystricognaths (*Cuscomys ashaninka* and *Salinoctomys loschalchalerosorum*) for which no information of ecology or body size was available.

We compared the list of species in NatureServe [39] with the updated IUCN database [40] to assess the extent to which changes in the taxonomic classification and/or the number of recognized mammal species may significantly influence our results (see S1 File for details). Primates were analysed at genus level throughout the present study (see below). Marsupials and hystricognaths were the two mammal groups most affected by taxonomic changes at species level. However, species richness patterns based on NatureServe data were strongly correlated with richness patterns estimated on the basis of IUCN [40] data (marsupials: Pearson correlation coefficient (r) with p-corrected for spatial autocorrelation [41] r = 0.922, p <.001; hystricognaths: r = 0.966, p = 0.005), also showing similar geographical trends (Figs. B and C in S1 File). We concluded that taxonomic updates to the problematic taxa in the database will not fundamentally change the results of this paper, although future studies are needed to overcome the deficiency of data on the geographical distribution and ecology of poorly documented species [40] that may potentially influence the estimation of ecological diversity.

We divided the New World into equal-area grid cells of 110 km x 110 km using an equal area Mollweide projection in ArcGis 9.2 [42]; coastal cells that included < 50% of land surface were excluded. We recorded the presence/absence of each species in each cell to define the extent of occurrence of each mammal group; for each mammal group, we retained only those cells with more than one mammal species.

### Choice of ecological attributes

Throughout the present study, we used the expression “ecological diversity” as an approximation to functional diversity, to mean the variety of attributes related to what organisms “do” in their habitat, so as to adopt a more flexible definition of functional diversity that does not require the association of attributes with ecosystem function [6]. To assess biologically meaningful relationships between taxonomic richness (TR), functional diversity (ED) and phylogenetic diversity (AvPD) we estimated functional and phylogenetic diversity using indices based on averages that do not increase with species richness [4].

Data on ecological attributes were compiled to represent different kinds of resource used by species and what species do to acquire them (body mass, home range size, resting or nesting site, substrate use, diet, activity cycle and group size) on the basis of 80 published sources, including books, articles and public databases (S2 File and S1 Dataset). These attributes represent the ecological relationships of mammal species with the environment and have been frequently used in previous analyses of mammal functional diversity (e.g., [1, 10, 23, 43]; S2 File); however, note that attributes may not be strictly comparable across different mammal groups (Tables H-M in S2 File, S1 Dataset).

This further justified taking different mammal groups separately for the analysis of diversity patterns.

Missing data at species level were replaced with data of congeneric species (as detailed in S2 File). For all mammal groups, we compiled the attributes at species level; however, the taxonomy of primates at this level is very unstable, since subspecies are frequently being elevated to the level of species [44, 45]. For primates, therefore, we compiled the attributes at species level (N = 128) but we did the analyses at genus level, (N = 18) as recommended for macroecological analyses [46].

### Estimation of ecological diversity

We estimated mean ecological diversity (ED) for each cell of the grid map using the Gower index (GI) (Gower, 1971); the mean Gower index (ED) indicates the mean dissimilarity between pairs of coexisting species [47].

Before the application of the Gower index, we applied weights to different attributes to ensure that all ecological aspects contributed equally to ED calculation [6]; Table G in S2 File shows a hypothetical example showing the effects of weighting attributes on the Gower index calculation). The coding and weighting values (W_i_) for different types of attributes (i) used for each mammal group are given in Tables H-M in S2 File.

For any pair *(j-k)* of mammal species, the Gower Index (GI_jk_), which ranges from 0 (complete similarity between species pairs) to 1 (complete dissimilarity between species pairs), was calculated as:

$$GI_{jk}=\frac{\sum W_{ijk}S_{ijk}}{\sum W_{ijk}}$$ (1)

Where:

S_ijk_ is the partial similarity coefficient of attribute i for the j-k pair of species.

W_ijk_ is the weight of attribute i for the j-k pair of species. W_ijk_ = 0 if species j and k cannot be compared for attribute i because either the value of this attribute for species j (X_ij_) or for species k (X_ik_) is unknown (i.e. non-available data for any attribute in any species that is compared).

For quantitative and ordinal attributes:

$$S_{ijk}=\frac{\left|X_{ij}-X_{ik}\right|}{\text{max}\{X_{i}\}-\text{min}\{X_{i}\}}$$

For the symmetric binary attributes (sensu [48]):

$$\begin{array}{l} S_{ijk}=0\;if\;X_{ij}=X_{ik}=1\\ S_{ijk}=0\;if\;X_{ij}=X_{ik}=0\\ S_{ijk}=1\;if\;X_{ij}\neq X_{ik} \end{array}$$

For the asymmetric binary attributes (sensu [48]):

S_ijk_ formula is the same as for symmetric binary attributes, but:

$$\;W_{ijk}=0\;if\;X_{ij}=X_{ik}=0$$

For nominal attributes:

$$\begin{array}{l} S_{ijk}=0\;if\;X_{ij}=X_{ik}\\ S_{ijk}=1\;if\;X_{ij}\neq X_{ik} \end{array}$$

For each cell, we computed GI_jk_ for each combination of species pairs. Then we summed up the values of all combinations of species pairs and divided this number by the total number of species pairs in each cell, to produce the mean GI. The mean Gower index was our estimation of ecological diversity (ED) per cell. It has the property of being independent of the number of species being compared, so it can be used to compare values across grid cells with different species richness.

We also estimated the coefficient of variation in ED (ED_cv_) as,

$$ED_{cv}=\frac{GI_{std}*\text{100}}{meanGI}$$ (2)

Where *meanGI* is the mean Gower Index for each cell and *GI*_std_ is its standard deviation.

The calculations were performed using R project, package FD [48, 49].

### Estimation of phylogenetic diversity

We classified mammal species (genera for primates) according to the mammal phylogeny of [50]. Species not included in this mammal supertree (marsupials: 2, xenarthrans: 0, artiodactyls: 3, carnivorans: 3, hystricognaths: 31, primates: 45) were appended taxonomically as basal polytomies within the clades representing the corresponding genera; inconsistencies in species names between the phylogeny and NatureServe list were resolved using [51].

For the calculation of AvPD, we adopted a pairwise distance approach similar to the estimation of Average Taxonomic Distance [52], adapted to phylogenetic distances. We first calculated the phylogenetic distance between each pair of taxa (species for all mammal groups, genera for primates) recorded in each cell on the grid map, taken as the sum of the branch lengths connecting each pair of taxa over the mammal phylogeny. We then added up the phylogenetic distances between all pairs of species (or genera for primates) present in each cell and divided this number by the total number of species (or genera) pairs recorded in each cell to obtain a measure of AvPD. Indices calculated by averaging data on pairwise comparisons have the property of being unbiased by species richness [53]. Calculations were performed using the picante package in R [54].

### Estimation of taxonomic richness

Taxonomic richness (TR) was the total number of species (marsupials, xenarthrans, artiodactyls, carnivorans, hystricognath rodents) or genera (primates) present in each cell of the grid map.

### Data analysis

For each mammal group, the overall associations between TR, ED, and AvPD were examined using bivariate scatterplots. We used the Pearson Correlation Coefficient (r) with p-corrected for spatial autocorrelation [41] to evaluate magnitude of associations.

We mapped TR, ED, ED_cv_ and AvPD to assess geographical patterns on a continental scale. We used Geographically Weighted Regression analysis (GWR) to assess regional variability in the extent to which AvPD accounted for variation in ED, while controlling for differences in TR. To analyze geographical variability in model predictions and diversity associations, we mapped AvPD beta coefficients, local R^2^ and residuals obtained from GWR as applied in SAM software [55], using adaptive Gaussian kernels with bandwidths that included the nearest 10% neighbours. Wherever positive beta coefficients were obtained, this supported the prediction that closely related species are more similar in their ecology than less closely related species; the higher the R^2^ value obtained, the better the capacity of AvPD to account for ED.

Safi et al. [1] analysed the dispersion (= residuals) around the global relationship between functional and phylogenetic diversity in order to identify areas showing comparable levels of phylogenetic distance, where the amount of functional similarity is lower or greater than expected. We used a similar approach to examine the spatial distribution of residuals of ED after fitting the GWR models (with AvPD and TR included as predictors in the models) to look for regions with higher or lower ecological diversity than others. Higher residual values than expected from AvPD (in the presence of TR) indicate areas that accumulated an excess of ED. In contrast, lower residual values than expected from AvPD indicated areas where ecological diversity was limited (deficit of ED).

## Results

### Geographical gradients in different components of diversity

**Fig 1:**
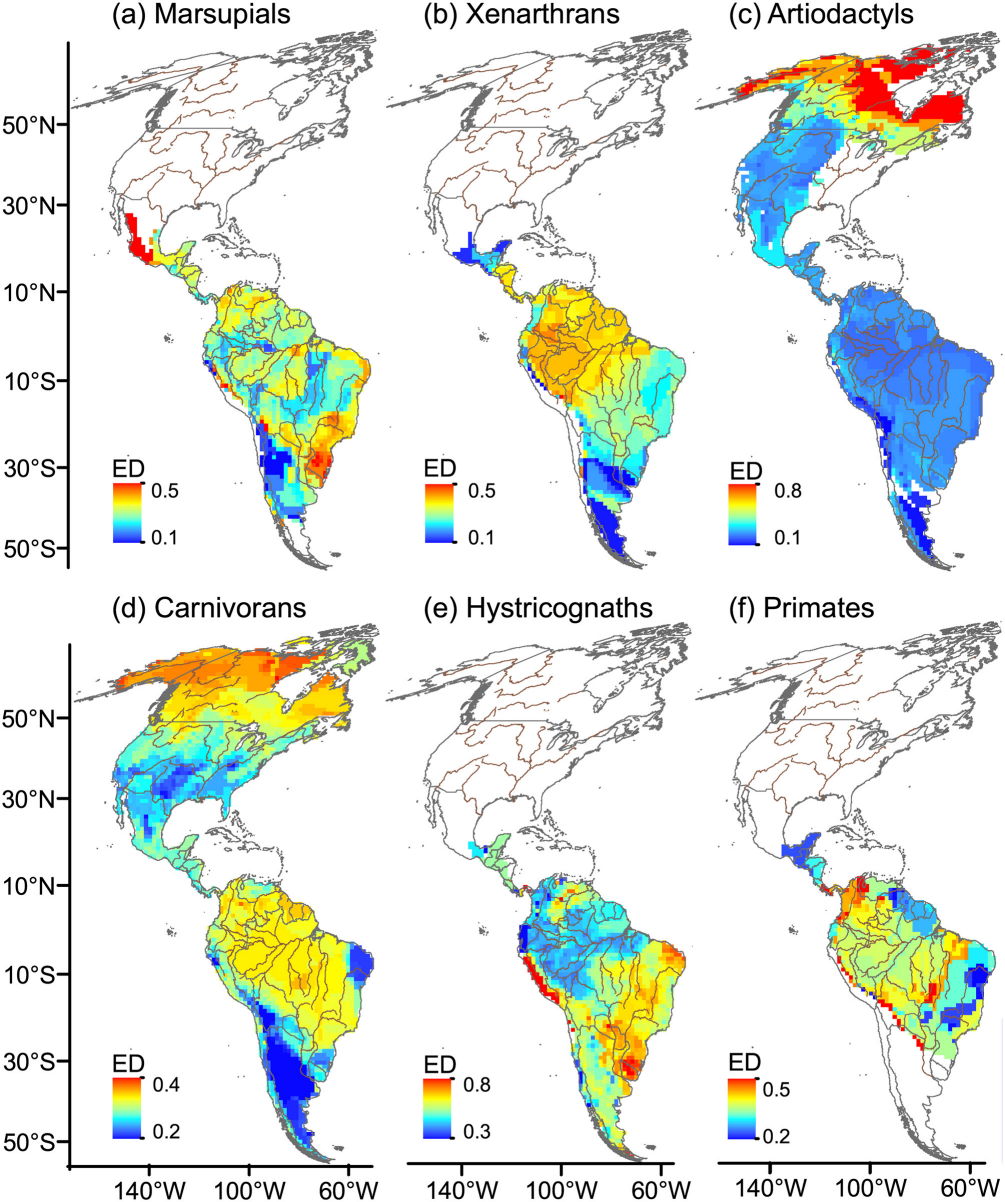
Spatial variation in ecological diversity (ED). ED was estimated by summing the Gower index values that quantified the pairwise dissimilarity between co-occurring species in each cell, and dividing this number by the total number of pairs in each 110 x 110 km-cell of the grid map. Scale units run between 0 (maximum similarity) and 1 (minimum similarity). For primates, all calculations were based on genus pairs. Rivers are drawn as brown lines and national borders are shown in grey. Maps are in Mollweide equal-area projection.

**Fig 2:**
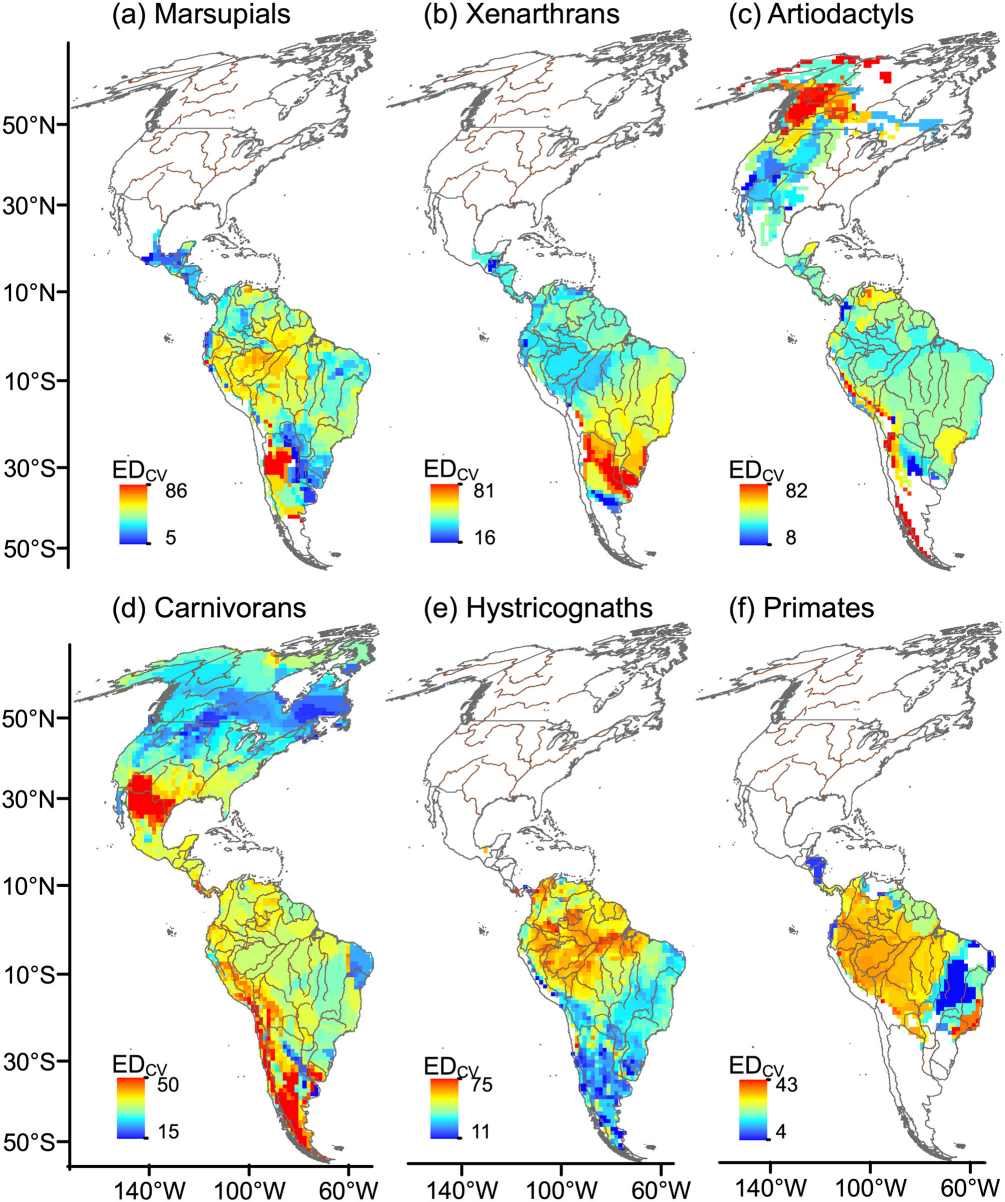
Spatial variation in the coefficient of variation of ecological diversity (ED_cv_). ED_cv_ was calculated as the ratio of standard deviation in Gower Index and mean Gower Index for each 110 x 110 km-cell of the grid map and was reported as a percentage. Rivers are drawn as brown lines and national borders are shown in grey. Maps are in Mollweide equal-area projection.

**Fig 3:**
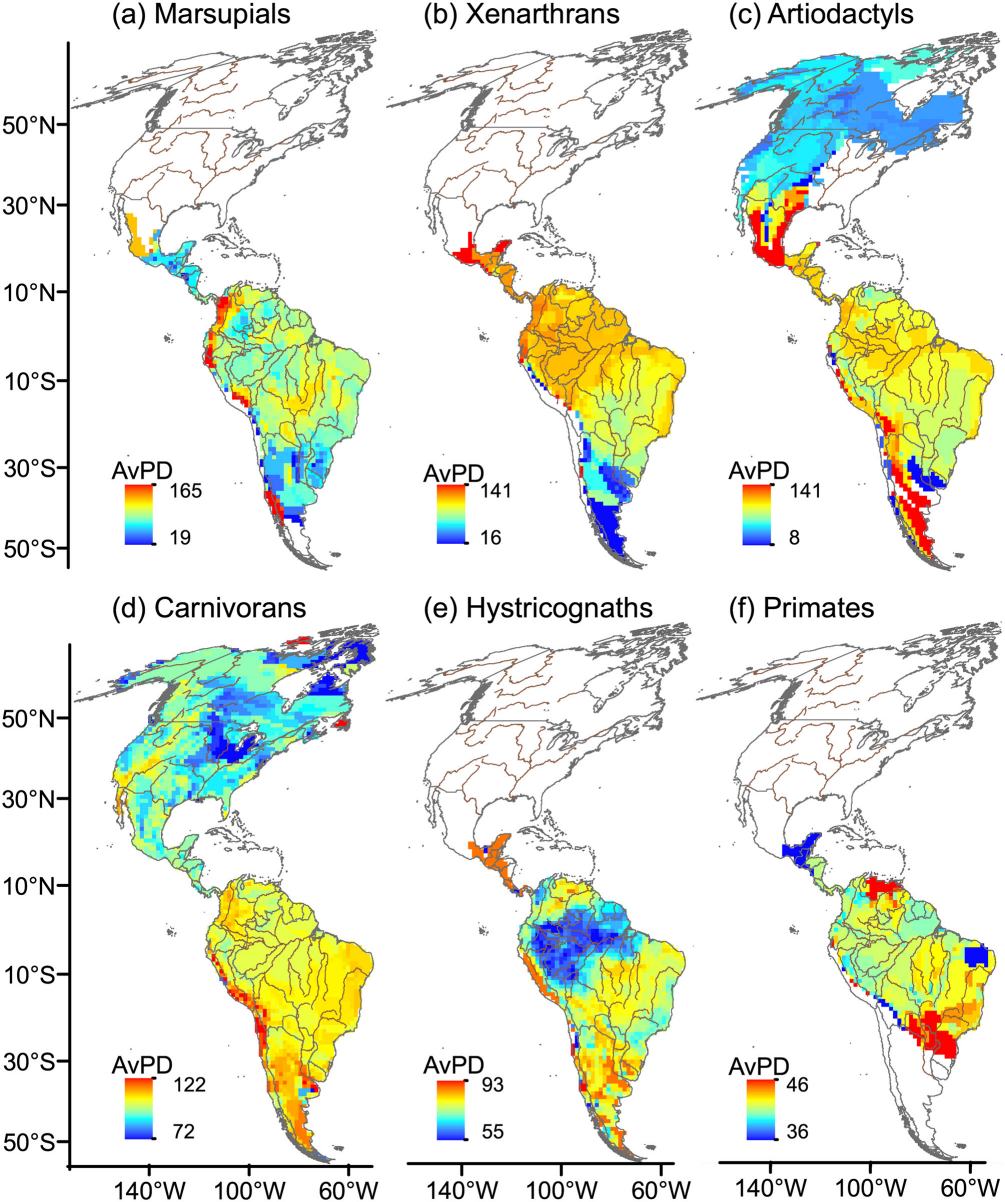
Spatial variation in phylogenetic diversity (AvPD). AvPD was estimated as the sum of the branch lengths in the phylogeny connecting all pairs of coexisting taxa (= species for all mammal groups, genera for primates), and dividing this number by the total number of pairs in each 110 x 110 km-cell of the grid map. Rivers are drawn as brown lines and national borders are shown in grey. Maps are in Mollweide equal-area projection.

**Fig 4:**
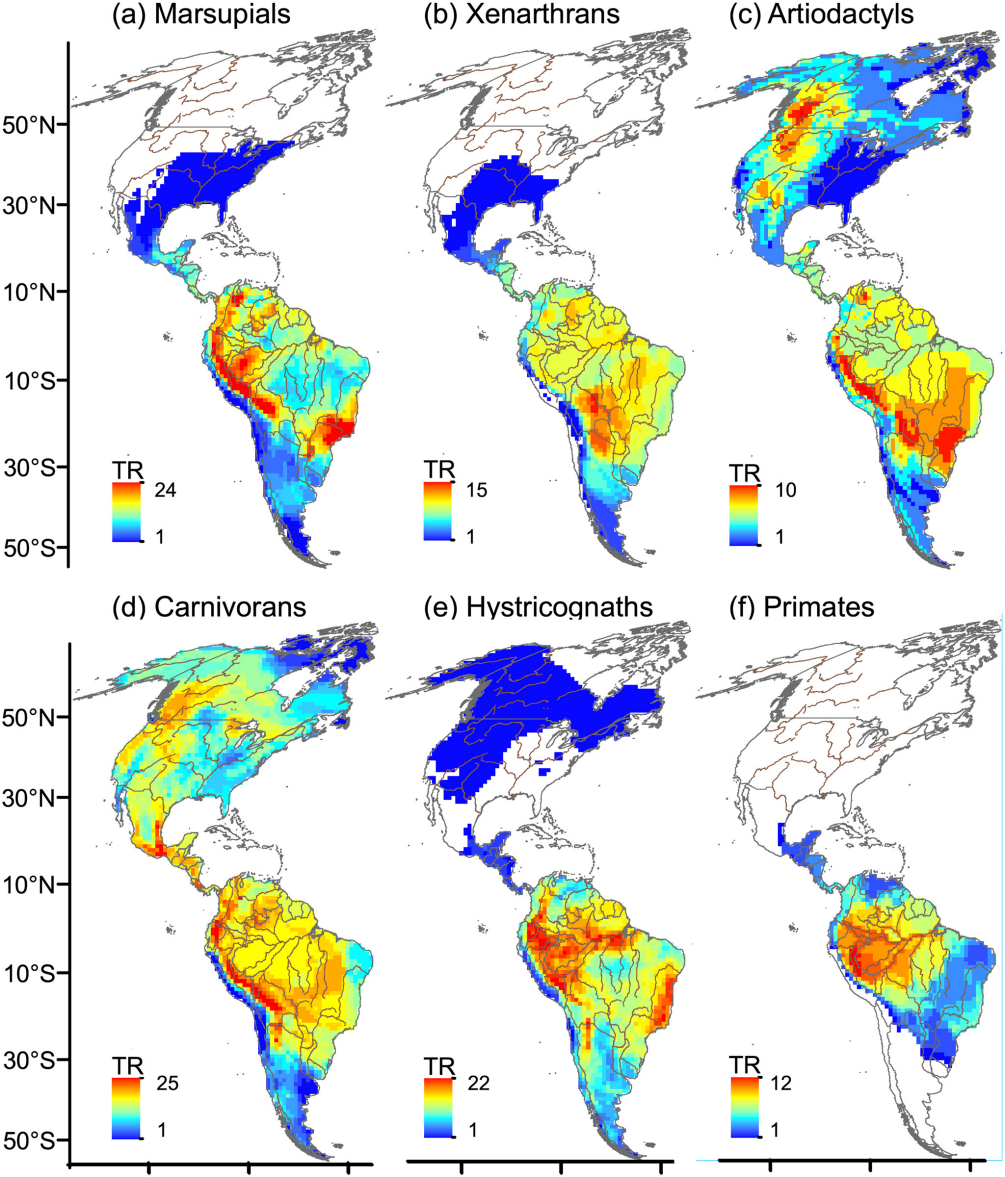
Spatial variation in taxonomic richness (TR). TR was calculated as the total number of species counted in each 110 x 110 km-cell of the grid map. Genera richness was calculated for primates. Rivers are drawn as brown lines and national borders are shown in grey. Maps are in Mollweide equal-area projection.

Spatial variation in ED, ED_cv_, AvPD and TR (Figs 1–4) was highly structured over the continental surface. In Suth America, marsupials, xenarthrans, carnivorans and primates (Fig 1a and 1b and 1d–1f) showed high ED in tropical rainforests within the Amazon Basin, and a tendency for ED to decrease towards southern temperate latitudes (except primates). Artiodactyls (Fig 1c) showed low ED over most of the continent. Hystricognaths differed in that they showed increased ED in arid tropical and temperate southern regions of South America (Fig 1e). In North America, carnivorans and artiodactyls showed a reverse latitudinal gradient of decreasing ED towards tropical latitudes (Fig 1c and 1d).

In areas of high ED and low ED_cv_ all species pairs were different from each other; these areas are found in northern North America for carnivorans (compare Figs 1d and 2d), tropical rainforest in the Amazon Basin for xenarthrans (Figs 1b and 2b), the Peruvian coast for hystricognaths (Figs 1e and 2e), and Uruguayan savannas and Caatinga for marsupials (Figs 1a and 2a) and hystricognaths (Figs 1e and 2e).

Marsupials tended to show increasing AvPD towards the western slope of the Andes in Colombia, Ecuador and northern Peru, along the eastern slopes in southern Peru and along both eastern and western slopes in central Chile and Argentina (Fig 3a). The AvPD values for xenarthrans peaked at the northern limit of their distribution, tending to be high within the tropical rainforests in the Amazon Basin, western slopes of the Andes in Colombia and Ecuadorian and Brazilian coastal rainforests, while they decreased in arid regions of central Brazil and towards the south, with minimum values in Patagonia (Fig 3b). The AvPD of artiodactyls, carnivorans and hystricognaths increased in arid regions and open habitats along the Andes of Peru, Bolivia and Chile, and towards southern South America (Fig 3c–3e). In North America, AvPD followed the classical latitudinal gradient of decreasing diversity at high latitudes, showing more marked changes for artiodactyls than for carnivorans (Fig 3c and 3d).

All mammal groups showed an increase in TR towards tropical latitudes in South America (Fig 4); in North America, carnivorans and artiodactyls showed richness peaks at temperate latitudes (Fig 4c and 4d).

### Overall statistical associations between different components of diversity

**Fig 5:**
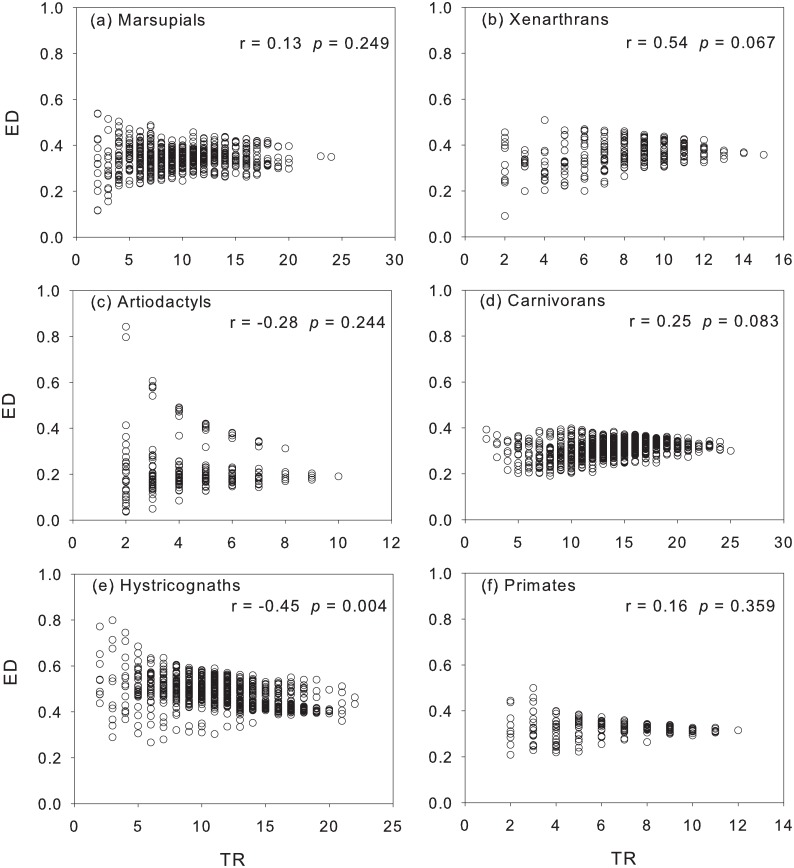
The relationship between ED and TR examined out of the geographical context. ED is ecological diversity and TR is taxonomic richness.

**Fig 6:**
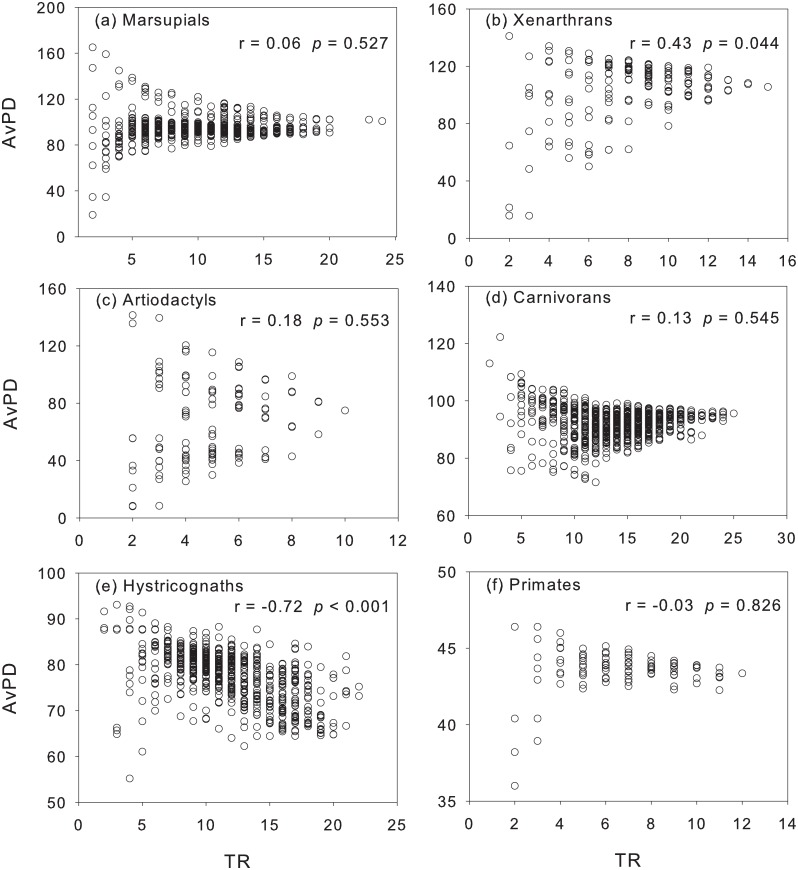
The relationship between phylogenetic diversity (AvPD) and taxonomic richness (TR). The relationship is examined out of the geographical context.

**Fig 7:**
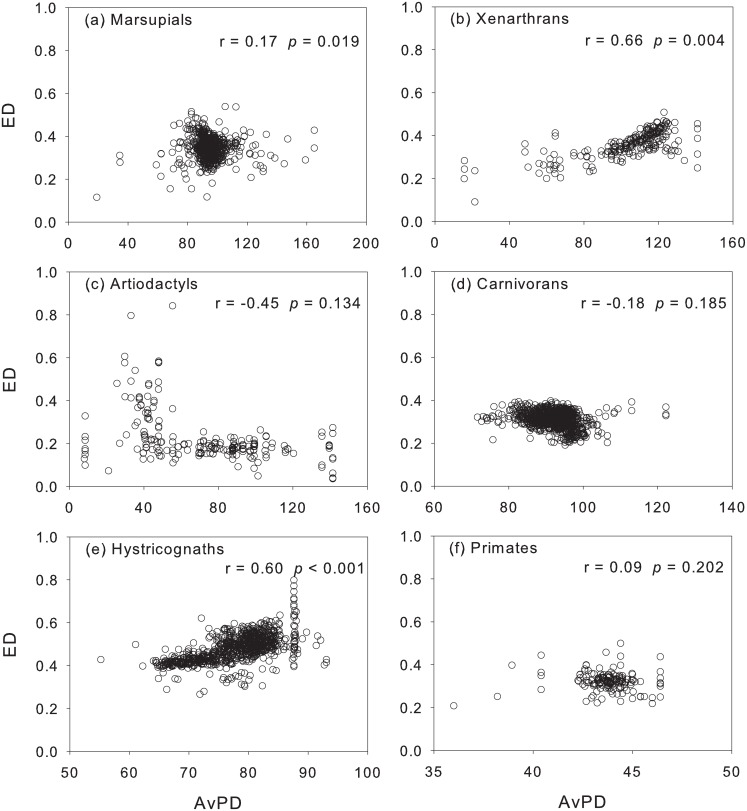
Relationship between ecological diversity (ED) and phylogenetic diversity (AvPD). The relationship is examined out of the geographical context.

ED and AvPD showed no significant correlation with TR (p > 0.05) in most of the mammal groups (marsupials, artiodactyls, carnivorans, primates) (Figs 5 and 6), suggesting funnel-like relationships that are typical of dissimilarity metrics based on averaging [5, 49]; xenarthrans showed that AvPD tends to increase with TR (Fig 6b). Hystricognaths were an exception in that they showed a significant decrease in ED and AvPD with TR (Figs 5e and 6e). ED increased significantly with AvPD in marsupials, xenarthrans, and hystricognaths; the trend was negative (but weak) in carnivorans and artiodactyls (p > 0.05), and primates showed no association (Fig 7).

### Regional associations between different components of diversity

**Fig 8:**
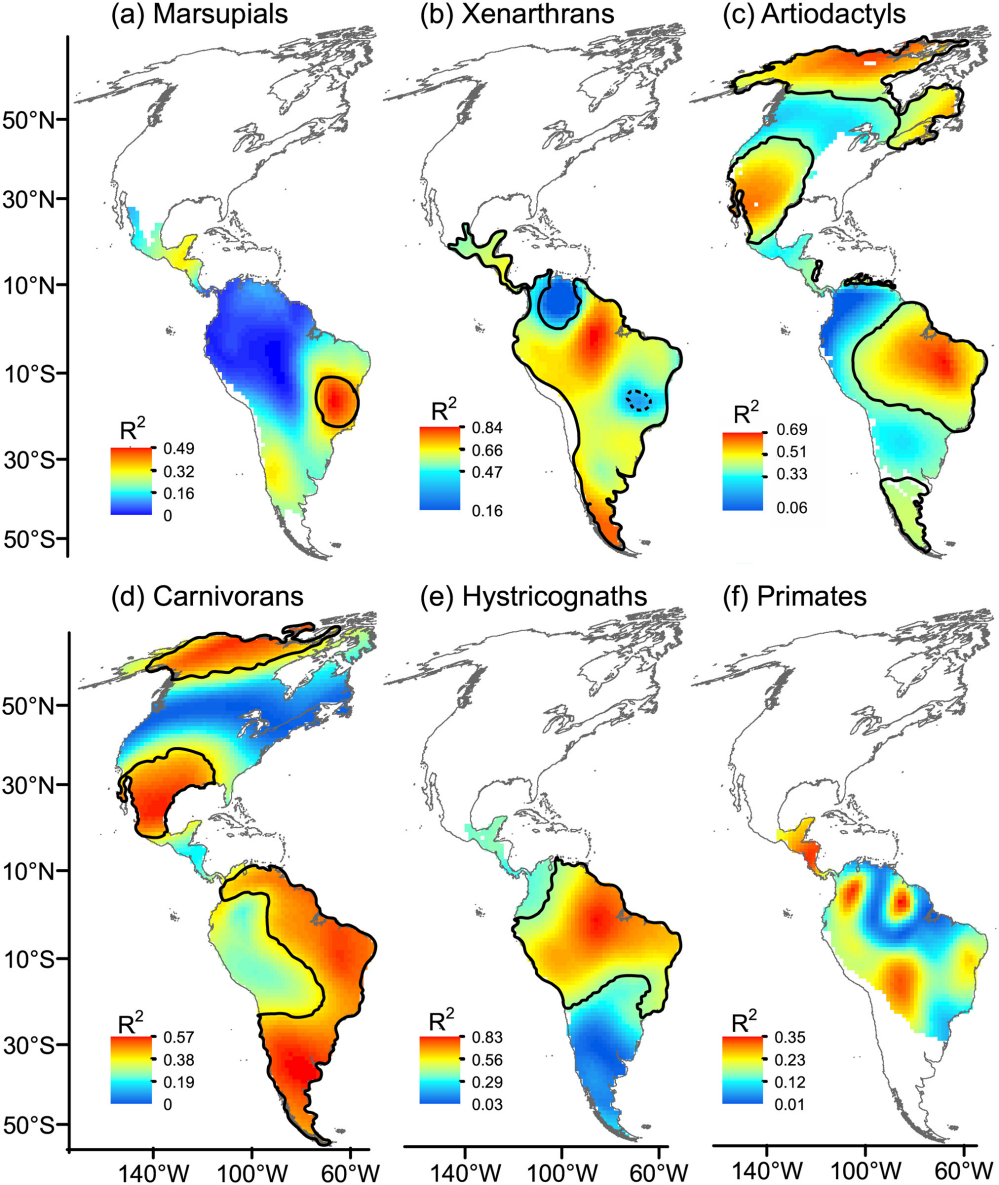
Local coefficients of determination (R^2^) obtained from geographically weighted regression (GWR). The local R^**2**^ coefficients indicate the proportion of variation in ecological diversity (ED) that was accounted for by phylogenetic diversity (AvPD) while controlling for differences in taxonomic richness (TR). Closed black lines denote areas of R^2^ higher than 0.4. The area enclosed by a dotted line in xenarthrans showed R^2^ equal to or lower than 0.4. Maps are in Mollweide equal-area projection.

TR and AvPD accounted for a significant proportion of the mean spatial variation in ED in tropical regions, including the Amazon Basin (xenarthrans, artiodactyls, hystricognaths), arid regions of northeast Brazil, northeastern coastal regions of South America (marsupials, xenarthrans, artiodactyls, carnivorans, hystricognaths), at the transition between the Neotropical and Nearctic regions, i.e., México and southern USA (artiodactyls, carnivorans) and in temperate regions at high latitudes in North America (artiodactyls and carnivorans) (Fig 8a–8f). In temperate regions of southern South America, the proportion of the variation in ED accounted for by the spatial variation in TR and AvPD was considerably lower than 40% in marsupials and hystricognaths and higher than 40% in xenarthrans, artiodactyls and carnivorans (Fig 8).

### Regional associations between ED and AvPD

**Fig 9:**
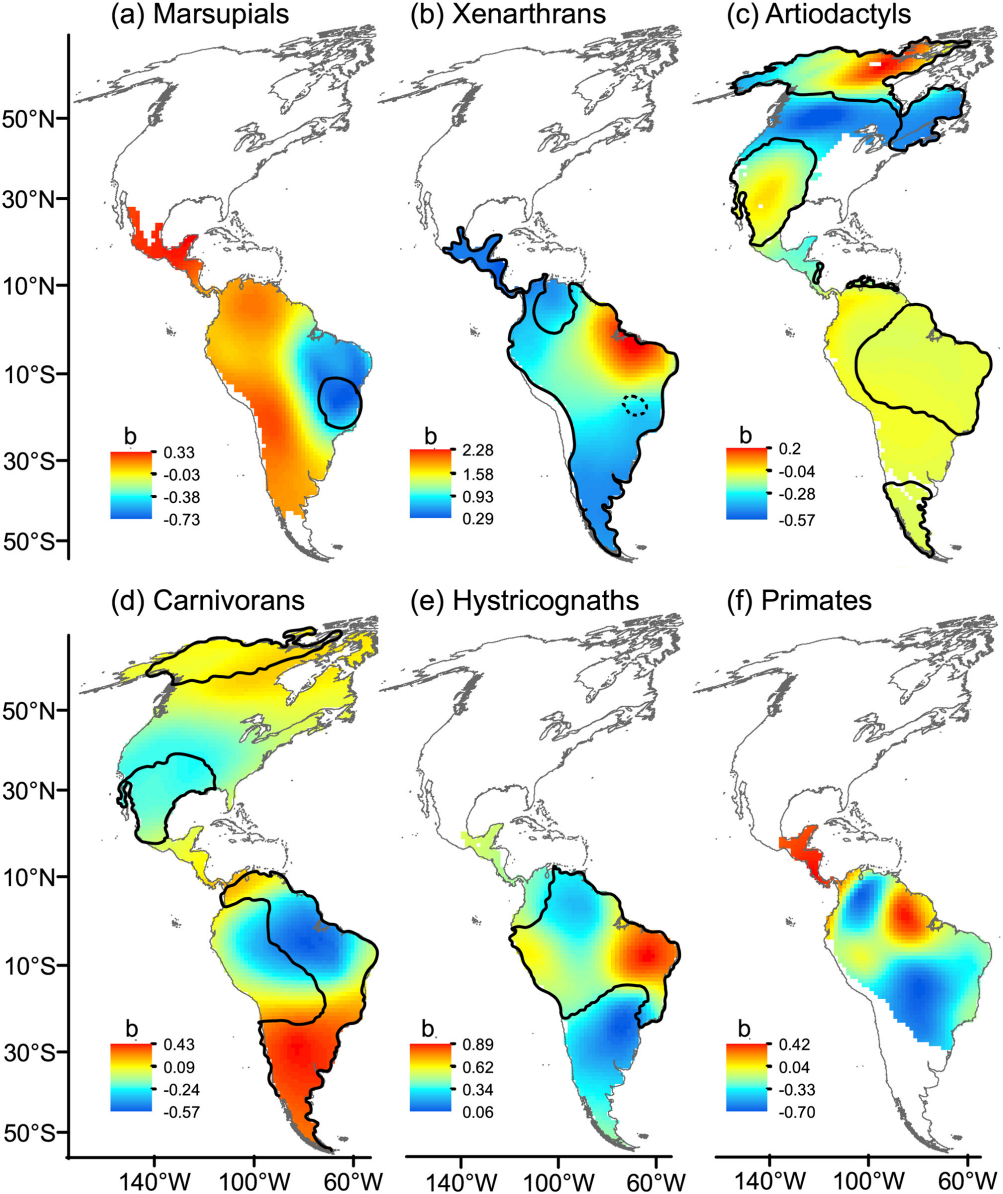
Local beta coefficients (b) of the effect of phylogenetic diversity (AvPD) on ecological diversity (ED). Local beta coefficients were obtained from geographically weighted regression (GWR) controlling for differences in taxonomic richness (TR). Closed black lines denote areas with coefficients of determination (R^2^) higher than 0.4. The area enclosed by a dotted line in xenarthrans showed R^2^ equal to or lower than 0.4. Maps are in Mollweide equal-area projection.

After controlling for TR, ED increased with AvPD over almost all the Neotropics in xenarthrans, (Fig 9b); marsupials also showed a widespread positive association but the trend changed to negative in arid regions of northeast Brazil. Hystricognaths contrasted with marsupials, showing the highest positive ED-AvPD association in that arid region. Hystricognaths and primates also showed an increase in ED with AvPD in the Amazon Basin and Central America (primates, Fig 9f). A positive association between ED and AvPD was also observed in temperate regions at high latitudes in North America (artiodactyls, Fig 9c) and in southern South America (carnivorans, Fig 9d); however, ED also decreased with AvPD (R^2^ > 0.40) for some mammal groups in some arid regions (marsupials: arid regions of northeast Brazil (Fig 9a), carnivorans: the Amazon Basin, and deserts and xeric shrublands in Mexico and USA (Fig 9d).

### Identification of areas with deficit of ED

**Fig 10:**
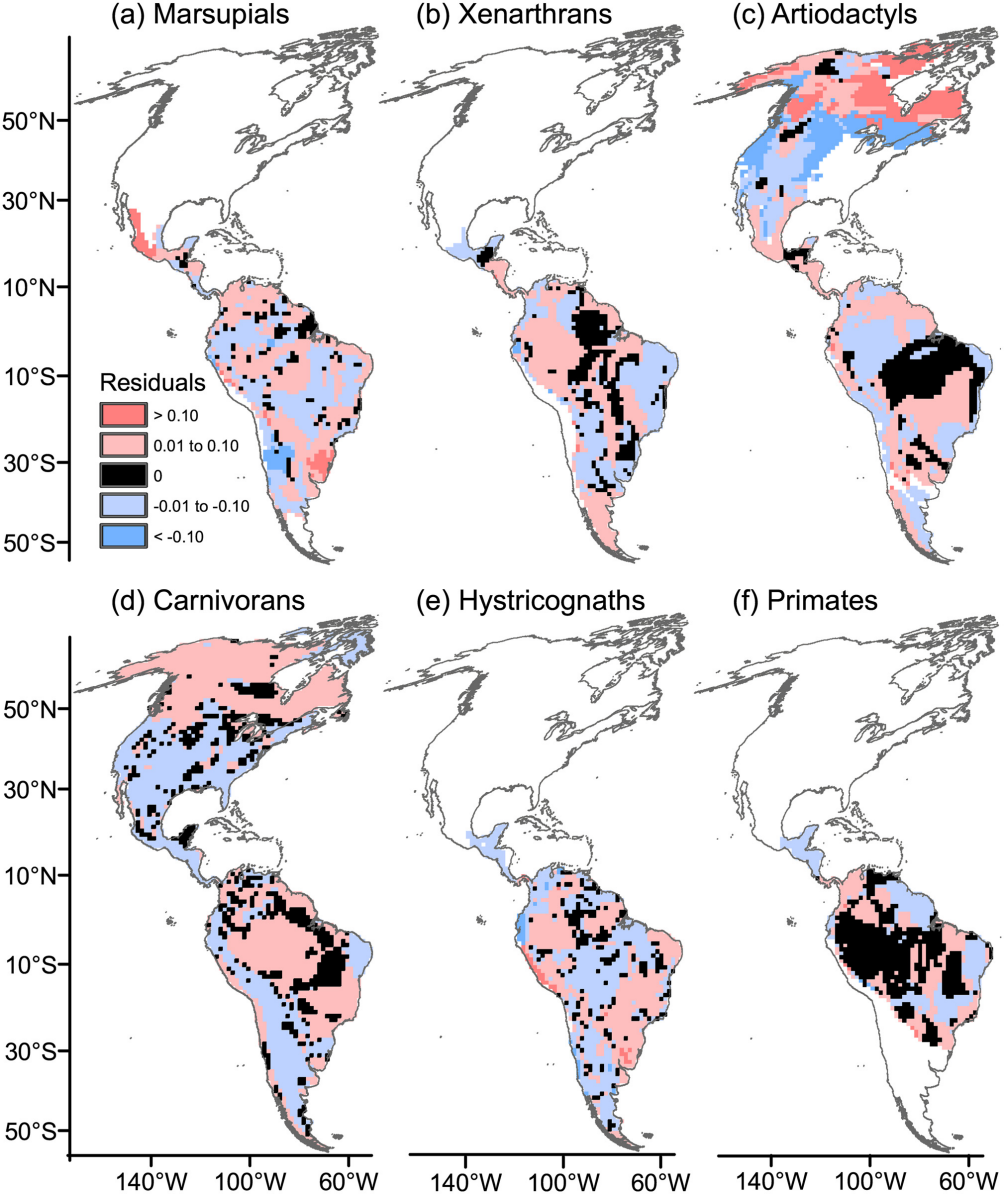
The residuals of ecological diversity (ED) obtained from geographically weighted regression (GWR). The residuals were obtained after fitting the models where phylogenetic diversity (AvPD) accounted for variation in ED, while controlling for differences in taxonomic richness (TR). Maps are in Mollweide equal-area projection.

Residuals of ED after model fitting (Fig 10) were spatially structured; however, at tropical latitudes in the Neotropics, taxa of tropical affinity (marsupials, xenarthrans, hystricognaths and primates) showed a patchy structure of regions with higher ED than predicted by TR and AvPD, interspersed with zones of ED deficit. Artiodactyls and carnivorans showed lower ED than predicted by TR and AvPD in some tropical arid regions in North and South America, including deserts and xeric shrublands in Mexico and southern USA (Fig 10c and 10d). All mammal groups except hystricognaths showed deficit of ED in the arid Caatinga of NE Brazil.

### Identification of areas with excess of ED

The extreme deserts along the Peruvian Pacific coast (Sechura and Atacama deserts) showed excess of ED for carnivorans, hystricognaths and partially also for artiodactyls (Fig 10c–10e); the arid Caatinga also showed excess of ED, but only for hystricognaths.

Artiodactyls and carnivorans distinguished the temperate high latitudes in North America as having higher ED than predicted from AvPD and TR (Fig 10c and 10d). In southern South America, artiodactyls and carnivorans (Fig 10c and 10d) distinguished between the western humid Subantarctic forests with higher ED than predicted by TR and AvPD and the Patagonian steppes in Argentina with ED deficit; hystricognaths showed a deficit of ED in both subregions (Fig 10e).

## Discussion

### Taxonomic richness is a poor predictor of other components of mammal diversity

Our study on South American mammals supports that species richness captures only a small amount of information about the other components of diversity [1, 16, 23, 56, 57]; only in hystricognaths did we find that increasing TR was associated with a significant decrease in ED and AvPD. Other studies using different diversity metrics also found a negative or weak relationship between richness and other components of diversity (e.g., evenness of functional groups: [43]; size diversity: [18]; morphological disparity: [58]; trait diversity: [10]). We quantified ED and AvPD using indices that are not associated with TR, thus the regions of high species richness in hystricognaths might be associated with assembly processes that led to the coexistence of closely related species that are ecologically similar.

The general lack of congruence between high TR and high ED or AvPD regions reinforces the idea that different components of biological diversity must be considered in the establishment of conservation priorities [16, 19, 23, 57, 59–61]. Furthermore, areas of high ED and low ED_cv_ indicated where high disparity is a common phenomenon between pairs of coexisting species, and thus these areas may be of particular interest for the conservation of diversity in different mammal groups and ecosystem function: northern North America (carnivorans), tropical rainforest in the Amazon Basin (xenarthrans), the Peruvian coast (hystricognaths), and Uruguayan savannas and Caatinga (marsupials and hystricognaths).

### Why different components of diversity are not well correlated with one another: the latitudinal gradient revisited

On a continental scale, TR patterns confirmed the classical latitudinal gradient of increasing richness towards the tropics in South America; however, artiodactyls and carnivorans also showed high richness in temperate latitudes in North America, which is probably associated with the coexistence of species of different biogeographic affinity (i.e., species belonging to evolutionary lineages of Eurasian affinity coexist with species of autochthonous lineages at temperate latitudes in North America, see [62]), and with the tendency of the number of carnivoran species from families of temperate origin to increase as temperature decreased in North America [24].

The geographical variation in ED and AvPD showed considerable variability across different mammal groups in North and South America, which explains the weak overall statistical associations between the different components of diversity, as previously found in other studies [17, 18, 23, 63, 64]. We found a reverse latitudinal gradient of high ED in temperate-cold regions of North America for carnivorans and artiodactyls, and in South America for hystricognaths, suggesting that the hypothesis of temperate-cold zones harbouring more functional and morphological diversity in mammals than tropical ones (e.g., [1, 18]) cannot be generalized for all taxa. Marsupials and xenarthrans showed a latitudinal decrease in ED and AvPD, although not in parallel with the latitudinal richness gradient.

Hystricognaths, artiodactyls, and carnivorans also showed high AvPD outside the tropics. In artiodactyls and hystricognaths the occurrence of low ED and AvPD in high richness regions may reflect major radiations of only few lineages and in situ speciation, with no further successful colonization by other lineages of the same taxon [5]. Overall, our results suggest that patterns based on components of diversity that take species identities into account differ strongly from the classical pattern of latitudinal decrease in species numbers, in agreement with other studies on mammals using diversity measures that are independent from species numbers (body mass variation: [23]; morphological diversity: [18]).

Our study suggested that the general expectation of a positive relationship between ecological diversity and phylogenetic diversity might be overstated [15]. When the ED-AvPD relationship was examined through simple scatterplots (i.e., out of the geographical context), we did not find evidence to support a general positive association between ED and AvPD consistently across different mammal groups, i.e. although the xenarthrans and hystricognaths showed overall significant strong positive AvPD-ED associations, there was a weak positive association in marsupials. In terms of geographic space, AvPD positively influenced ED for some groups and in some regions: South America for xenarthrans, the tropics for hystricognaths, northern North America for newcomers (artiodactyls and carnivorans), and southern South America for carnivorans. ED and AvPD were not associated in primates.

### South American mammals of tropical affinity do not show a predominance of ED deficit within tropical latitudes

Taxa of tropical affinity (marsupials, xenarthrans, hystricognaths and primates) highlighted the tropics as a complex mosaic of regions with excess of ED interspersed with zones of deficit of ED, thus rejecting our original prediction (Fig 10). This finding suggests that niche conservatism may be not predominant over niche evolution in accounting for high diversity within the tropics. Safi et al. [1] observed a tropical-temperate gradient of ecological diversity, suggesting the predominance of functional diversity deficits within the tropics when all terrestrial mammals were analysed on a global scale. Although our results suggested a different pattern for South American mammals, the studies are not necessarily contradictory. On the one hand, the pattern found when all mammals were combined may be the result of a few mammal groups that contain a high number of species, and consequently have a strong influence on global results [24, 65]. For instance, we considered only hystricognath rodents, but not other rodent clades with a high number of species in America (i.e., Castorimorpha-Myomorpha and Sciuromorpha); however, including all rodent clades could influence overall patterns based on all mammals taken together. On the other hand, the use of different modelling techniques may also account for differences between the present study and Safi et al. (1), because we used GWR leading to beta coefficients that varied regionally, whereas Safi et al. [1] fit an overall global model. Also, given that climatic parameters accounted for significant portions of variation in ED on a global scale [1], the presence of zones with ED excess or deficit may ultimately result from the environmental effect underlying the regional variability of ED.

### Newcomers from North America show ED patterns consistent with the niche conservatism hypothesis

Artiodactyls and carnivorans are representative of newcomer lineages that migrated into South America during the GABI [36, 38]. As predicted, the pattern in the ED residuals for carnivorans might have resulted from the occurrence of niche conservatism along the eastern flank of the Andes, over most of a region that formed an open corridor linking the Patagonian steppes and grasslands of southern Argentina with the grasslands of Colombia, and continuing northward across the Panamanian land bridge to southeastern North America [38] (see maps of biomes and main climates in America in Fig. A in S3 File). The pattern seen in the ED residuals is consistent with the idea that carnivorans were probably able to expand from North America southwards by tracking this open corridor, while being conservative in their ecological requirements. Artiodactyls confirmed the prediction of niche conservatism in arid regions of North America, but not so clearly in South America.

### South American mammals suggest niche evolution could have occurred at both tropical and extra-tropical latitudes

The completion of the Antarctic ice sheet in Late Miocene promoted the general cooling and drying of the Patagonian region and the formation of the Argentinean pampas, in association with a major shift in vegetation [32]. This led to the predominance of savannas and grasslands that triggered diversification (e.g., living euphractine armadillos in the pampas and savannas of southern South America [32]), increasing the ecological diversity of open habitats in southern South America. Consistent with this account, our study showed an increase in ED for xenarthrans at high latitudes in South America.

The general cooling and drying of southern South America was also associated with the appearance of evolutionary novelty (the appearance of hypsodont dental morphology in hystricognaths (abrocomids, octodontines and ctenomyines [31]) and the extinction of lineages with primitive molars, which led to the subsequent appearance of marked desert specializations among octodontines in coincidence with a profound global Late Pliocene cooling and drying event [31, 66]. These changes could explain the occurrence of high ED in association with high AvPD of hystricognaths in open habitats of southern South America and in arid regions of tropical South America (i.e., deserts along the Peruvian coast, and arid regions in the Caatinga and Cerrado in northeast and southeast Brazil). We did not find evidence of excess of ED for hystricognaths in southern South America, which could reflect the presence of a high number of *Ctenomys* species that appeared to be ecologically similar, although many aspects of their ecology are still unknown. Our results suggest that stronger selection pressures for hystricognaths might have occurred in the extreme desert regions of tropical South America than in southern South America.

Marsupials, the remaining mammal group of tropical affinity that extend into extra-tropical latitudes, did not show an increase (or excess) in ED towards high latitudes in southern South America. Thus, our study did not support the idea of the predominance of niche evolution at high latitudes in South America. In contrast, we did find support for niche evolution in carnivorans and artiodactyls at high latitudes in North America. Interestingly, carnivorans and artiodactyls also suggested that niche evolution could possibly have been involved in the occupation of cold and humid Andean forest at high latitudes in southern South America, which suggests that cold-temperate humid forests along the southern Andes may represent “new” environmental conditions for those mammal lineages with affinity for open habitat conditions.

We conclude that different components of diversity are not surrogates for one another, since a decoupling between functional and phylogenetic components of diversity might have been promoted by the occurrence of niche evolution in well-delimited regions, i.e., in association with arid environments at tropical latitudes and cold-temperate climates at extra-tropical latitudes. On the other hand, South American mammals suggest that niche conservatism is not predominant over niche evolution at tropical latitudes and thus cannot be considered the primary, single explanation to account for the latitudinal diversity gradient.

## Supporting Information

S1 Dataset (XLS)

S1 File Influence of taxonomic changes on the assessment of mammal distributional patterns. (DOC)

S2 File Choice of attributes and estimation of ecological diversity. (DOC)

S3 File The geographical distribution of highlands, major rivers, biomes and main climates in America. (DOC)
