# Correction: Ecological Diversity in South American Mammals: Their Geographical Distribution Shows Variable Associations with Phylogenetic Diversity and Does Not Follow the Latitudinal Richness Gradient

**DOI:** 10.1371/journal.pone.0134651

**Published:** 2015-07-29

**Authors:** The PLOS ONE Staff

## Notice of Republication

This article was republished on July 15, 2015, due to errors in Figs. 1–4 and 8–10. Please download this article again to view the correct version. The originally published, uncorrected article and the republished, corrected article are provided here for reference.

## Supporting Information

S1 File Originally published, uncorrected article. (PDF)

S2 File Republished, corrected article. (PDF)
