# Supplementary material for: Ecological Diversity in South American Mammals: Their Geographical Distribution Shows Variable Associations with Phylogenetic Diversity and Does Not Follow the Latitudinal Richness Gradient
Source: PLoS One. 2015 Jun 8;10(6):e0128264. doi: 10.1371/journal.pone.0128264 (PMC4460121; doi:10.1371/journal.pone.0128264)
Supplement: S1 File — (DOC) [file pone.0128264.s002.doc]

**S1 File. Influence of taxonomic changes on the assessment of mammal distributional patterns**

We used the IUCN 2014 database [1] to identify taxonomic changes or new species descriptions that may have caused changes in species’ geographical distributions. We identified the IUCN updates that may have resulted in our estimations of species richness (based on NatureServe 2003 [2]) being underestimated (Figure Aa in S1 File), overestimated (Figure Ab in S1 File) or with weak or no changes (Figure Ac in S1 File). Primates were analysed at genus level throughout the present study and were thus excluded from the NatureServe 2003 [2] – IUCN 2014 [1] comparison throughout.

*IUCN* [1] *updates that may have caused our estimations of local richness based on NatureServe* [2] *to be underestimated (Figure Aa in S1 File)*: There were a total of 58 species recognized in IUCN 2014 [1] that were not recorded in NatureServe 2003 [2] (marsupials: N = 23, artiodactyls: N = 1, carnivorans: N = 1, hystricognath: N = 33). In addition, *Thylamys fenestrae* recently split from *T. pallidior* and was thus listed in IUCN 2014 [1] but not in NatureServe 2003 [2]; this taxonomic change may have resulted in an underestimation of richness towards the southern distribution limit of *T. pallidior,* where the two species are sympatric.

*IUCN* [1] *updates that may have caused our estimations of local richness based on NaturServe* [2] *to be overestimated (Figure Ab in S1 File)*: 4 species recognized in NatureServe 2003 [2] were not recorded in IUCN 2014 [1] (artiodactyls: N = 1, hystricognaths: N = 3). There were formerly partially sympatric “species” that IUCN 2014 [1] recognized as a single valid species (e.g. marsupials: *Marmosops noctivagus* includes *M. dorothea,* and *Monodelphis dimidiata* now includes *M. sorex*). We observed this kind of taxonomic update five times in hystricognaths, involving changes in the geographical distribution of eleven species recorded in NatureServe [2].

*IUCN* [1] *updates with weak or no effect on our local estimations of species richness (Figure Ac in S1 File)*: Some taxonomic identities recorded in NatureServe 2003 [2] as species have been split into multiple species with parapatric/allopatric distributions IUCN 2014 [1], although these taxonomic changes should not have an effect on local richness patterns. For example, *Mazama nemorivaga* and *M. gouazoubira* are considered two valid parapatric species in IUCN 2014 [1]; however, NatureServe [2] included *M. nemorivaga* within *M. gouazoubira*. This kind of split was found in marsupials (N = 1), artiodactyls (N = 2) and hystricognaths (N = 6). Similarly, some parapatric (or allopatric) species recorded in NatureServe 2003 [2] (e.g. *Conepatus leuconotus* and *Conepatus mesoleucus*) are now considered as a single species, and this unified the geographical ranges of the two formerly recognized species. This kind of update affected the taxonomic distributions of species of marsupials (N = 4), carnivorans (N = 5) and hystricognaths (N = 3).

Marsupials and hystricognaths were the two mammal groups most affected by the taxonomic updates at species level; however, their species richness patterns estimated from data of either NatureServe 2003 [2] or IUCN 2014 [1] did not differ substantially (Figures B and C in S1 File).

**A Fig.** **Schematic diagram of the kinds of taxonomic update in IUCN 2014 [1] (IUCN-14) that could affect the geographical distribution of species recognized in NatureServe 2003 [2] (NS-03).** The circles represent geographic ranges.


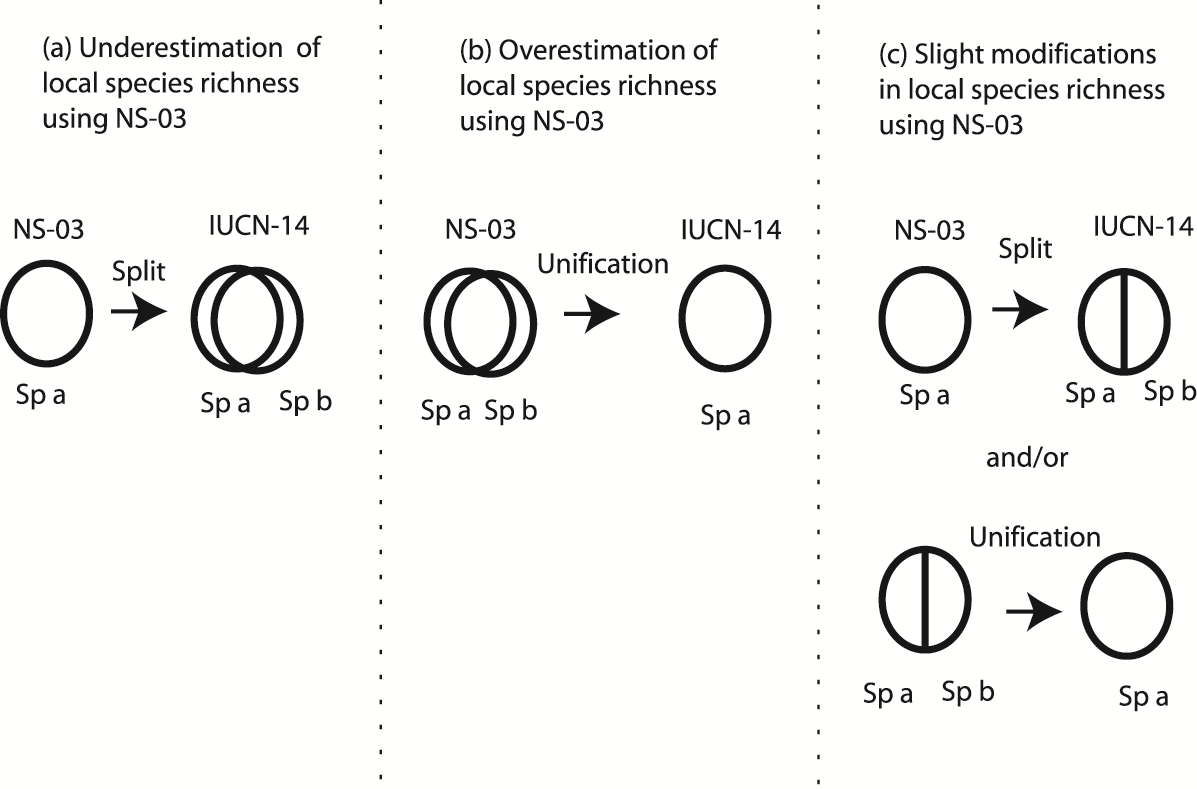


**B Fig.** **Species richness (SR) patterns estimated on the basis of NatureServe 2003 [2] (NS-03) and IUCN 2014 [1] (IUCN-14) databases for marsupials.**

**C Fig.** **Species richness (SR) patterns estimated on the basis of NatureServe 2003 [2] (NS-03) and IUCN 2014 [1] (IUCN-14) databases for hystricognaths.**

**References**
