# Supplementary material for: Ecological Diversity in South American Mammals: Their Geographical Distribution Shows Variable Associations with Phylogenetic Diversity and Does Not Follow the Latitudinal Richness Gradient
Source: PLoS One. 2015 Jun 8;10(6):e0128264. doi: 10.1371/journal.pone.0128264 (PMC4460121; doi:10.1371/journal.pone.0128264)
Supplement: S2 File — (DOC) [file pone.0128264.s003.doc]

**S2 File. Choice of attributes and estimation of ecological diversity**

1. ***Choice of ecological attributes: their ecological relevance***

*Body mass:* Size and body mass are important predictors of many physiological and ecological traits in mammals. They are considered good predictors of the ecological niche of species (e.g., [1]). The variability in body mass is considered a proxy of functional diversity due to its relationship with ecosystem functioning; for example, small mammals are generally insectivorous or seed dispersers [2]. Within the groups of mammals analyzed here, body mass is related to the a) diet requirements of the species (e.g., marsupials: [3]; primates: [4]) , b) the energy spent in the maintenance of vital functions (e.g., metabolic rate in marsupials: [3], xenarthros: [5]), c) the type of locomotion or maximum running speed (e.g., marsupials: [3, 6]; artiodactyls: [6], [7]; carnivorans: [6-8]; rodents and primates: [6]), d) the use of the substrate (e.g., vertical stratum in marsupials: [3]; carnivorans: [8]), e) the mechanisms of torpor (e.g., marsupials: [3]), and f) home range size (e.g., carnivorans: [9]; primates: [10]).

*Horizontal space use*: This was represented by the home range size, which is related to the area that mammals use in their foraging and reproduction activities. Home range size is closely related to mammals’ energy requirements [11] and is also associated with dispersal distances [12].

*Group size*: This was represented by the number of individuals in a group. This information was not available for rodents so we used an indicator of gregariousness (whether groups have 1-2 individuals or more). Group size is generally considered an anti-predator response, since larger groups reduce the risk of predation per capita, by increasing collective vigilance and predator detection, or by collective defense ([13] and references therein). In artiodactyls, group size is related to anti-predator behavior, since as the body size of artiodactyls increases the relative maximum running speed decreases, so then the anti-predatory response is an increase in group size [6]. However, group size is also related to other ecological and social aspects of mammals. In rodents, group size is related to the risk of predation and the behaviour of burrow digging [13]. In primates group size is related to social system, diet, use of arboreal vertical stratum and home range size [14].

*Activity cycle*: This was represented by quantification of diurnal and nocturnal activity. In rodents, crepuscular activity is also taken into account. The active period is related to resource use and it is influenced by physiological constraints. Phylogeny also plays a role in the determination of activity cycles (e.g. [15]).

*Diet*: This is the main indicator of resources used. For each mammal group, it was represented here by several dietary attributes that reflect amplitude of diet. In xenarthrans the specialization of feeding on ants and termites was also taken into account, since it is an important variable that describes the different feeding types and allows us to distinguish between the diets of different species of armadillos [5].

*Substrate use*: The use of different types of substrate provides a large amount of ecological information [16] and is a direct indicator of the relationship between mammals and their environment. It is also related to locomotive adaptations in mammals, although not synonymous [16]. Here, the substrate used by mammals was represented by the different media they utilize, including arboreal, terrestrial and underground. In xenarthrans, the relationship with the underground substrate was taken into account, considering their burrow digging ability and their tendency to be fossorial (Table I). In primates, the different heights of the arboreal substrata that they utilize were taken into account (Table M). This is important because it reflects details of their feeding strategy, such as their ability to reach fruits growing higher in the canopy layer (e.g., [17]). Also, in primates, the use of different tree layer heights is related to the maintenance of some biological interactions, for example the ability to form mixed troops with other primate species or to coexist in the same space with other species (e.g., [17]).

*Site for rest or nesting:* It is important to take the substrate used by mammal species for passive activities into account to understand more about their relationship with their environment [16]. Passive activities include resting, sleeping, grooming and rearing young [16]. This ecological aspect was represented here by the quantification of sites used by mammals for nesting, sleeping or resting. In addition, the re-utilization of sites abandoned by other species was considered, including the use of abandoned bird nests (marsupials, Table H) or beavers (carnivorans, Table K).

Data on ecological attributes for each mammal group were extracted from several references (Tables A-F).

We applied a substitution method based on the calculation of mean values of congeneric species to estimate missing values of quantitative attributes. For qualitative attributes missing values were substituted with the most frequent attribute state recorded in the congeneric species. The percentages of estimated data were: marsupials 45.7%, xenarthrans 17%, artiodactyls 23.4%, carnivorans 16.8%, and rodents 45.8%. After these estimations, the percentages of remaining missing values (NA) were low: marsupials 1.33%, xenarthrans: 5.33%, artiodactyls 0 %, carnivorans 2.4 %, rodents 3.7%). For primates we compiled the attributes at species level but we performed the analyses at genus level. The percentage of NA data at genus level was 1.8%.

A Table. References used in the elaboration of the ecological database of marsupials

| Reference | Body  mass | Activity cycle | Diet | Substrate  utilization | Place for rest  or nesting |
| --- | --- | --- | --- | --- | --- |
| [18] | x | x | x | x |  |
| [19] |  | x | x | x | x |
| [20] |  | x | x | x | x |
| [21] |  | x | x | x | x |
| [22] | x |  |  |  |  |
| [23] |  |  |  |  | x |
| [24] |  | x | x | x | x |
| [25] |  |  |  |  |  |
| [26] |  | x | x | x | x |
| [27] |  | x | x | x | x |
| [28] |  | x | x | x | x |
| [29] |  |  |  |  | x |
| [30] |  | x | x | x | x |
| [31] |  |  | x | x | x |

B Table. References used in the elaboration of the ecological database of xenarthrans

| Reference | Body mass | Horizontal  use of space | Activity  cycle | Diet | Substrate  utilization |
| --- | --- | --- | --- | --- | --- |
| [32] | x |  |  |  |  |
| [33] |  | x |  |  |  |
| [19] | x | x | x | x | x |
| [20] | x | x | x | x | x |
| [21] |  |  | x |  | x |
| [22] | x | x | x |  |  |
| [5] | x | x | x | x | x |
| [24] | x |  | x | x | x |
| [25] |  |  |  |  |  |
| [26] | x | x | x | x | x |
| [27] |  | x | x | x | x |
| [28] |  |  | x | x | x |
| [24] |  |  |  | x |  |
| [25] | x |  |  |  |  |
| [36] |  |  |  |  | x |
| [30] | x | x | x | x | x |
| [31] |  |  | x | x | x |

C Table. References used in the elaboration of the ecological database of Artiodactyls

| Reference | Body  mass | Horizontal use of space | Group size | Activity  cycle | Diet |
| --- | --- | --- | --- | --- | --- |
| [19] |  | x | x | x | x |
| [20] |  | x | x | x | x |
| [21] |  |  | x | x | x |
| [37] |  |  | x |  |  |
| [22] | x | x | x | x |  |
| [38] |  | x |  |  |  |
| [24] |  | x | x | x | x |
| [25] |  | x |  | x |  |
| [39] |  |  |  | x |  |
| [40] |  | x |  |  |  |
| [26] |  | x |  | x | x |
| [27] |  | x | x | x | x |
| [28] |  | x | x | x | x |
| [41] | x |  |  |  |  |
| [29] |  | x |  |  |  |
| [30] |  | x | x | x | x |
| [31] |  |  | x | x | x |

D Table. References used in the elaboration of the ecological database of carnivorans

| Reference | Body  mass | Horizontal use of  space | Group  size | Activity  cycle | Substrate  use | Diet | Place for rest or nesting |
| --- | --- | --- | --- | --- | --- | --- | --- |
| [42] |  |  | x | x | x | x | x |
| [43] |  |  | x | x |  | x | x |
| [44] |  |  | x |  |  | x |  |
| [45] |  | x |  | x | x |  |  |
| [46] |  | x |  |  |  |  |  |
| [47] |  |  |  |  |  | x |  |
| [48] |  |  |  |  |  | x |  |
| [19] |  |  | x | x | x | x | x |
| [20] |  |  | x | x | x | x | x |
| [21] |  |  | x | x | x |  | x |
| [49] |  |  |  | x | x |  |  |
| [22] | x | x | x | x |  |  |  |
| [50] |  | x |  |  |  |  |  |
| [51] |  | x |  |  |  |  |  |
| [52] |  |  |  | x |  |  | x |
| [53] |  |  |  | x |  | x | x |
| [54] |  |  |  | x |  |  | x |
| [55] |  | x |  |  |  |  |  |
| [56] |  | x |  | x |  | x |  |
| [24] |  | x | x | x | x | x | x |
| [57] |  |  |  | x |  |  |  |
| [25] |  | x | x | x |  | x | x |
| [58] |  |  | x |  |  |  |  |
| [39] |  |  | x |  |  |  |  |
| [59] |  |  | x |  |  |  |  |
| [26] |  |  | x | x | x | x | x |
| [27] |  |  | x | x | x | x | x |
| [28] |  |  | x | x | x | x | x |
| [60] |  | x |  |  |  |  |  |
| [41] | x |  |  |  |  |  |  |
| [29] |  | x |  |  |  | x | x |
| [8] |  |  |  |  | x |  |  |
| [30] |  |  | x | x | x | x | x |
| [31] |  |  | x | x | x | x | x |
| [61] |  |  | x |  |  |  |  |
| [62] |  |  |  | x |  |  | x |
| [63] |  |  | x | x |  |  |  |
| [64] |  |  | x | x | x | x | x |
| [65] |  |  |  | x | x | x | x |

E Table. References used in the elaboration of the ecological database of hystricognath rodents

| Reference | Body  mass | Gregariousness | Activity  cycle | Diet | Place for rest or nesting  and  burrowing | Substrate  use/ locomotor adaptations |
| --- | --- | --- | --- | --- | --- | --- |
| [42] |  |  |  | x |  |  |
| [66] |  |  |  |  |  | x |
| [67] |  |  |  | x |  |  |
| [68] | x |  |  |  |  |  |
| [69] |  |  | x |  | x | x |
| [70] |  |  |  |  | x |  |
| [13] |  | x | x |  | x |  |
| [71] |  | x |  |  | x |  |
| [19] |  | x | x | x | x | x |
| [20] |  | x | x | x | x | x |
| [21] |  | x | x | x | x | x |
| [72] |  | x | x | x | x | x |
| [22] | x | x | x |  |  |  |
| [73] |  | x |  | x |  |  |
| [74] |  | x |  |  |  |  |
| [75] |  | x |  |  |  |  |
| [76] |  |  |  |  |  | x |
| [77] |  |  |  |  | x |  |
| [24] |  | x | x | x | x | x |
| [78] |  |  |  |  |  | x |
| [79] |  |  | x |  |  | x |
| [25] |  |  |  | x | x |  |
| [26] |  | x | x | x | x | x |
| [27] |  | x | x | x | x | x |
| [28] |  | x | x | x | x |  |
| [80] |  | x |  | x | x | x |
| [81] |  |  |  | x |  |  |
| [82] |  |  |  |  |  | x |
| [83] |  |  |  |  | x |  |
| [84] |  | x |  | x | x |  |
| [85] |  | x | x | x |  | x |
| [30] |  | x | x | x | x | x |
| [86] |  |  |  |  |  | x |
| [31] |  | x | x | x | x | x |

F Table. References used in the elaboration of the ecological database of primates

| Reference | Body size | Group size | Activity  cycle | Diet | Substrate  use |
| --- | --- | --- | --- | --- | --- |
| [87] |  | x |  | x |  |
| [88] |  | x |  | x |  |
| [19] | x | x | x | x | x |
| [20] | x | x | x | x | x |
| [21] | x |  | x | x | x |
| [89] |  | x | x | x |  |
| [22] |  |  |  |  |  |
| [90] |  | x |  |  |  |
| [24] | x | x | x | x | x |
| [25] |  |  |  | x |  |
| [26] | x | x | x | x | x |
| [27] |  |  | x | x | x |
| [28] |  |  |  |  |  |
| [4] |  |  |  | x |  |
| [17] | x | x | x | x | x |
| [41] | x |  |  |  |  |
| [91] | x |  |  |  |  |
| [92] |  |  |  |  |  |
| [30] | x |  | x | x | x |
| [31] |  |  |  |  |  |

G Table. The estimation of dissimilarity between a pair of hypothetical species using the Gower index [93], as applied with and without attribute weighting. The weights were assigned to ensure that the two ecological aspects, diet and activity cycle, have the same relevance in the calculation of Gower index. In this hypothetical example diet is represented by five attributes (seeds, leaves, fruits, invertebrates, vertebrates) whereas the activity cycle is represented by only two attributes (nocturnal, diurnal). Without weighting of attributes, the Gower index value is lower (0.29] because the two species share the same state for 5 out of the 7 attributes. In contrast, after weighting the attributes to ensure that each aspect adds up to 1, the Gower index value is 0.5 because the two species share their diet but differ in their activity cycle.

|  | Diet | | | | | Activity cycle | |  |
| --- | --- | --- | --- | --- | --- | --- | --- | --- |
|  | Seeds | Leaves | Fruits | Inverte  brates | Verte  brates | Diurnal | Nocturnal | Gower index  between  species A  and B |
| Species A | 1 | 1 | 1 | 1 | 1 | 0 | 1 |  |
| Species B | 1 | 1 | 1 | 1 | 1 | 1 | 0 |  |
| Without  weighting  of  attributes | - | - | - | - | - | - | - | 0.29 |
| With  weighting  of  attributes | 0.2 | 0.2 | 0.2 | 0.2 | 0.2 | 0.5 | 0.5 | 0.5 |

H Table. Codification and weighting of different types of attributes compiled for marsupials. Group size was not included because the individuals are solitary. Home range size was not included because there is not enough data available

| Ecological aspect | Attribute | Attribute  weight (W) | Type of attribute | Coding |
| --- | --- | --- | --- | --- |
| Body mass | Body mass | 1.00 | Quantitative |  |
| Activity cycle | Diurnal | 0.50 | Binary  asymmetric | Present = 1  Absent = 0 |
|  | Nocturnal | 0.50 | Binary asymmetric | Present = 1  Absent = 0 |
| Diet | Fruit | 0.20 | Binary asymmetric | Consumes = 1  Does not consume = 0 |
|  | Exudates | 0.20 | Binary asymmetric | Consumes = 1  Does not consume = 0 |
|  | Invertebrates | 0.20 | Binary asymmetric | Consumes = 1  Does not consume = 0 |
|  | Vertebrates | 0.20 | Binary asymmetric | Consumes = 1  Does not consume = 0 |
|  | Carrion | 0.20 | Binary asymmetric | Consumes = 1  Does not consume = 0 |
| Substrate use | Arboreal substrate | 0.33 | Binary asymmetric | Utilizes = 1  Does not utilize = 0 |
|  | Terrestrial substrate | 0.33 | Binary asymmetric | Utilizes = 1  Does not utilize = 0 |
|  | Semi aquatic substrate | 0.33 | Binary asymmetric | Utilizes = 1  Does not utilize = 0 |
| Site for rest or nesting | Hollow logs or trees | 0.25 | Binary asymmetric | Utilizes = 1  Does not utilize = 0 |
|  | Burrows | 0.25 | Binary asymmetric | Utilizes = 1  Does not utilize = 0 |
|  | Crevices | 0.25 | Binary asymmetric | Utilizes = 1  Does not utilize = 0 |
|  | Abandoned nests of birds | 0.25 | Binary asymmetric | Utilizes = 1  Does not utilize = 0 |
|  |  |  |  |  |

I Table. Codification and weighting of different types of attributes compiled for xenarthrans. Group size was not included because the individuals are solitary.

| Ecological aspect | Attribute | Attribute  weight (W) | Type of attribute | Coding |
| --- | --- | --- | --- | --- |
| Body mass | Body mass | 1.00 | Quantitative |  |
| Horizontal use of space | Home range size | 1.00 | Quantitative |  |
| Activity cycle | Diurnal | 0.50 | Binary asymmetric | Present = 1  Absent = 0 |
|  | Nocturnal | 0.50 | Binary asymmetric | Present = 1  Absent = 0 |
| Diet | Specialization in ants and termites | 0.12 | Ordinal | * Specialist. diet based on ants and termites = 4  * Consumes ants and termites in a high percentage = 3  * Consumes ants and termites in a medium to low percentage = 2  * Does not consume ants or termites = 1 |
|  | Invertebrates | 0.12 | Binary asymmetric | Consumes = 1  Does not consume = 0 |
|  | Vertebrates | 0.12 | Binary asymmetric | Consumes = 1  Does not consume = 0 |
|  | Carrion | 0.12 | Binary asymmetric | Consumes = 1  Does not consume = 0 |
|  | Seeds | 0.12 | Binary asymmetric | Consumes = 1  Does not consume = 0 |
|  | Fruits | 0.12 | Binary asymmetric | Consumes = 1  Does not consume = 0 |
|  | Leaves | 0.12 | Binary asymmetric | Consumes = 1  Does not consume = 0 |
|  | Tubers and roots | 0.12 | Binary asymmetric | Consumes = 1  Does not consume = 0 |
| Substrate use | Arboreal substrate | 0.25 | Binary asymmetric | Utilizes = 1  Does not utilize = 0 |
|  | Terrestrial substrate | 0.25 | Binary asymmetric | Utilizes = 1  Does not utilize = 0 |
|  | Burrow building | 0.25 | Binary asymmetric | Excavates burrows= 1  Does not excavate burrows = 0 |
|  | Fossoriality in armadillos | 0.25 | Ordinal | * Burrowers = 3  * Often digs but digging plays no essential part in their feeding strategy and are not burrowers = 2  * Cursorial life. non digger = 1 |

J Table. Codification and weighting of different types of attributes compiled for artiodactyls. Substrate use was not considered because there is almost no variability across species.

| Ecological aspect | Attribute | Attribute  weight (W) | Type of attribute | Coding |
| --- | --- | --- | --- | --- |
| Body mass | Body mass | 1.00 | Quantitative |  |
| Horizontal use of space | Home range size | 1.00 | Quantitative |  |
| Group size | Group size | 1.00 | Quantitative |  |
|  |  |  |  |  |
| Activity cycle | Diurnal | 0.50 | Binary asymmetric | Absent = 0 |
|  | Nocturnal | 0.50 | Binary asymmetric | Present = 1  Absent = 0 |
| Diet | Grass and/or forbs | 0.08 | Binary asymmetric | Consumes = 1  Does not consume = 0 |
|  | Cacti | 0.08 | Binary asymmetric | Consumes = 1  Does not consume = 0 |
|  | Fungi | 0.08 | Binary asymmetric | Consumes = 1  Does not consume = 0 |
|  | Tubers and roots | 0.08 | Binary asymmetric | Consumes = 1  Does not consume = 0 |
|  | Fruits | 0.08 | Binary asymmetric | Consumes = 1  Does not consume = 0 |
|  | Leaves of shrubs | 0.08 | Binary asymmetric | Consumes = 1  Does not consume = 0 |
|  | Nuts | 0.08 | Binary asymmetric | Consumes = 1  Does not consume = 0 |
|  | Seeds | 0.08 | Binary asymmetric | Consumes = 1  Does not consume = 0 |
|  | Invertebrates | 0.08 | Binary asymmetric | Consumes = 1  Does not consume = 0 |
|  | Vertebrates | 0.08 | Binary asymmetric | Consumes = 1  Does not consume = 0 |
|  | Flowers | 0.08 | Binary asymmetric | Consumes = 1  Does not consume = 0 |
|  | Lichen | 0.08 | Binary asymmetric | Consumes = 1  Does not consume = 0 |
|  | Carrion | 0.08 | Binary asymmetric | Consumes = 1  Does not consume = 0 |

K Table. Codification and weighting of different types of attributes compiled for carnivorans.

| Ecological aspect | Attribute | Attribute weight (W) | Type of attribute | Coding |
| --- | --- | --- | --- | --- |
| Body mass | Body mass | 1.00 | Quantitative |  |
| Horizontal use of space | Home range size | 1.00 | Quantitative |  |
| Group size | Group size | 1.00 | Quantitative |  |
| Activity cycle | Diurnal | 0.33 | Binary asymmetric | Present = 1  Absent = 0 |
|  | Nocturnal | 0.33 | Binary asymmetric | Present = 1  Absent = 0 |
|  | Crepuscular | 0.33 | Binary asymmetric | Present = 1  Absent = 0 |
| Substrate use | Terrestrial substrate | 0.25 | Binary asymmetric | Utilizes = 1  Does not utilize = 0 |
|  | Arboreal substrate | 0.25 | Binary asymmetric | Utilizes = 1  Does not utilize = 0 |
|  | Aquatic substrate | 0.25 | Binary asymmetric | Utilizes = 1  Does not utilize = 0 |
|  | Underground substrate | 0.25 | Binary asymmetric | Utilizes = 1  Does not utilize = 0 |
| Diet | Fruit | 0.17 | Binary asymmetric | Consumes = 1  Does not consume = 0 |
|  | Invertebrates | 0.17 | Binary asymmetric | Consumes = 1  Does not consume = 0 |
|  | Small and/ or medium vertebrates | 0.17 | Binary asymmetric | Consumes = 1  Does not consume = 0 |
|  | Large vertebrates | 0.17 | Binary asymmetric | Consumes = 1  Does not consume = 0 |
|  | Carrion | 0.17 | Binary asymmetric | Consumes = 1  Does not consume = 0 |
|  | Green material | 0.17 | Binary asymmetric | Consumes = 1  Does not consume = 0 |
| Site for rest or nesting | Burrows | 0.13 | Binary asymmetric | Utilizes = 1  Does not utilize = 0 |
|  | Rock crevices | 0.13 | Binary asymmetric | Utilizes = 1  Does not utilize = 0 |
|  | Hollow logs | 0.13 | Binary asymmetric | Utilizes = 1  Does not utilize = 0 |
|  | Under trees or shrub bases | 0.13 | Binary asymmetric | Utilizes = 1  Does not utilize = 0 |
|  | Vegetation at soil level | 0.13 | Binary asymmetric | Utilizes = 1  Does not utilize = 0 |
|  | Up in tree cavities or tree branches | 0.13 | Binary asymmetric | Utilizes = 1  Does not utilize = 0 |
|  | Under snow | 0.13 | Binary asymmetric | Utilizes = 1  Does not utilize = 0 |
|  | Abandoned beaver lodges | 0.13 | Binary asymmetric | Utilizes = 1  Does not utilize = 0 |

L Table. Codification and weighting of different types of attributes compiled for hystricognath rodents. Home range size was not included because there is not enough data available.

| Ecological aspect | Attribute | Attribute  weight (W) | Type of  attribute | Coding |
| --- | --- | --- | --- | --- |
| Body mass | Body mass | 1.00 | Quantitative |  |
| Group size | Gregariousness | 1.00 | Binary symmetric | One or two individuals = 1  More than two individuals = 0 |
| Activity cycle | Diurnal | 0.33 | Binary asymmetric | Present = 1  Absent = 0 |
|  | Nocturnal | 0.33 | Binary asymmetric | Present = 1  Absent = 0 |
|  | Crepuscular | 0.33 | Binary asymmetric | Present = 1  Does not present = 0 |
| Diet | Roots and tubers | 0.14 | Binary asymmetric | Consumes = 1  Does not consume = 0 |
|  | Cacti or succulents | 0.14 | Binary asymmetric | Consumes = 1  Does not consume = 0 |
|  | Leaves of shrubs or tress | 0.14 | Binary asymmetric | Consumes = 1  Does not consume = 0 |
|  | Aerial grasses | 0.14 | Binary asymmetric | Consumes = 1  Does not consume = 0 |
|  | Seeds | 0.14 | Binary asymmetric | Consumes = 1  Does not consume = 0 |
|  | Fruits | 0.14 | Binary asymmetric | Consumes = 1  Does not consume = 0 |
|  | Insects | 0.14 | Binary asymmetric | Consumes = 1  Does not consume = 0 |
| Site for rest or nesting and burrow digging | Burrow digging | 0.20 | Binary asymmetric | Excavates burrows = 1  Does not excavate burrows = 0 |
|  | Use burrows. excavated by the species and/or by other species | 0.20 | Binary asymmetric | Utilizes = 1  Dos not utilize = 0 |
|  | Trees | 0.20 | Binary asymmetric | Utilizes = 1  Dos not utilize = 0 |
|  | Vegetation or hollow logs | 0.20 | Binary asymmetric | Utilizes = 1  Dos not utilize = 0 |
|  | Rock crevices | 0.20 | Binary asymmetric | Utilizes = 1  Dos not utilize = 0 |
| Substrate use |  | 1.00 | Nominal | Arboreal substrate  Terrestrial substrate  Arboreal-Terrestrial substrate  Aquatic-Terrestrial substrate  Terrestrial-Underground substrate  Underground substrate |

M Table. Codification and weighting of different types of attributes compiled for primates. In primates, the attributes were compiled at species level but diversity patterns were analysed at genus level. For all types of attributes the value assigned to each genus was the mean of the species’ values and so all attributes at genus level were quantitative. Only the activity cycle remains as binary symmetric (sensu [94]) because there is only one genus (*Aotus*) composed of all nocturnal species and the remaining genera are all diurnal.

| Ecological aspect | Attribute | Attribute  weight (W) | Type of attribute at species level | Coding at  species level | Type of attribute at genus level |
| --- | --- | --- | --- | --- | --- |
| Body mass | Body mass | 1.00 | Quantitative |  | Quantitative |
| Group size | Group size | 1.00 | Quantitative |  | Quantitative |
| Horizontal use of space | Home range | 0.50 | Quantitative |  | Quantitative |
|  | Day or night range | 0.50 | Quantitative |  | Quantitative |
| Activity cycle | Diurnal/  nocturnal | 1.00 | Binary symmetric | Diurnal = 1  Nocturnal = 0 | Binary symmetric |
| Diet | Seeds | 0.14 | Binary asymmetric | Consumes = 1  Does not consume = 0 | Quantitative |
|  | Exudates | 0.14 | Binary asymmetric | Consumes = 1  Does not consume = 0 | Quantitative |
|  | Leaves | 0.14 | Binary asymmetric | Consumes = 1  Does not consume = 0 | Quantitative |
|  | Invertebrates | 0.14 | Binary asymmetric | Consumes = 1  Does not consume = 0 | Quantitative |
|  | Flowers | 0.14 | Binary asymmetric | Consumes = 1  Does not consume = 0 | Quantitative |
|  | Vertebrates | 0.14 | Binary asymmetric | Consumes = 1  Does not consume = 0 | Quantitative |
|  | Nectar | 0.14 | Binary asymmetric | Consumes = 1  Does not consume = 0 | Quantitative |
| Substrate use | Terrestrial substrate (Frequently descends to the ground) | 0.25 | Binary asymmetric | Utilizes = 1  Does not utilize = 0 | Quantitative |
|  | Low strata in arboreal substrate | 0.25 | Binary asymmetric | Utilizes = 1  Does not utilize = 0 | Quantitative |
|  | Medium strata in arboreal substrate | 0.25 | Binary asymmetric | Utilizes = 1  Does not utilize = 0 | Quantitative |
|  | High strata in arboreal substrate | 0.25 | Binary asymmetric | Utilizes = 1  Does not utilize = 0 | Quantitative |

**References**

*(The asterisk indicates references used in the elaboration of the ecological data base)*

1. Eisenberg JF. The mammalian radiations: a study in evolution and adaptation. London: Athlone Press; 1981.

2. Fritz SA and Purvis A. Phylogenetic diversity does not capture body size variation at risk in the world's mammals. Proc R Soc Lon B. 2010;277: 2435-2441.

3. Jones M, Dickman CR and Archer M. Predators with pouches: the biology of carnivorous marsupials. Ligare: Csiro Publishing; 2003.

4.* Rosenberger AL. Evolution of feeding niches in New World monkeys. Am J Phys Anthropol. 1992;88: 525-562.

5.* Montgomery GG. The evolution and ecology of armadillos, sloth, and vermilinguas. Washington and London: Smithsonian institution press; 1985

6. Iriarte-Díaz J. Differential scaling of locomotor performance in small and large terrestrial mammals. J Exp Biol. 2002;205: 2897-2908.

7. Christiansen P. Locomotion in terrestrial mammals: the influence of body mass, limb length and bone proportions on speed. Zool J Linn Soc. 2002;136: 685-714.

8.* Van Valkenburgh B. Locomotor diversity within past and present guilds of large predatory mammals. Paleobiology. 1985;11: 406-428.

9. Lindstedt SL, Miller BJ and Buskirk SW. Home range, time, and body size in mammals. Ecology. 1986;67: 413-418.

10. Milton K and May ML. Body weight, diet and home range area in primates. Nature. 1976;259: 459-462.

11. Kelt DA and Van Vuren D. Energetic constraints and the relationship between body size and home range area in mammals. Ecology. 1999;80: 337-340.

12. Bowman J, Jaeger JAG and Fahrig L. Dispersal distance of mammals is proportional to home range size. Ecology. 2002;83: 2049-2055.

13.* Ebensperger LA and Blumstein DT. Sociality in New World hystricognath rodents is linked to predators and burrow digging. Behav Ecol. 2006;17: 410-418.

14. Terborgh J and Janson CH. The socioecology of primate groups. Annu Rev Ecol Syst. 1986;17: 111-136.

15. Roll U, Dayan T and Kronfeld-Schor N. On the role of phylogeny in determining activity patterns of rodents. Evol Ecol. 2006;20: 479-490.

16. Miljutin A. Substrate utilization and feeding strategies of mammals: description and classification. Estonian J Ecol. 2009;58: 60–71.

17.* Rowe N. The pictorial guide to the living primates. New York: Pogonios Press; 1996.

18.* Birney EC and Monjeau JA. Latitudinal variation in South American marsupial biology. In: Jones M, Dickman C and A. M, editors. Predators with Pouches The biology of carnivorous marsupials. Csiro Publishing; 2003. p. 297-331

19.* Eisenberg JF. Mammals of the neotropics. The northern neotropics: Panama, Colombia, Venezuela, Guyana, Suriname, French Guiana. Chicago and London: The University of Chicago Press; 1989.

20.* Eisenberg JF and Redford KH. Mammals of the neotropics. The central neotropics: Ecuador, Peru, Bolivia, Brazil. Chicago and London: The University of Chicago Press; 1999.

21.* Emmons LH. Neotropical rainforest mammals. Chicago and Londres: Chicago University Press; 1990

22.* Jones KE, Bielby J, Cardillo M, Fritz SA, O'Dell J, et al. PanTHERIA: a species-level database of life history, ecology, and geography of extant and recently extinct mammals. Ecology. 2009;90: 2648-2648.

23.* Martin GM and Sauthier DEU. Observations on the captive behavior of the rare Patagonian opossum *Lestodelphys halli* (Thomas, 1921)(Marsupialia, Didelphimorphia, Didelphidae). Mammalia. 2011;75: 281-286.

24.* Nowak RM. Walker’s mammals of the world. Baltimore and London: The Johns Hopkins University Press; 1991.

25.* Parera A. Los mamíferos de la Argentina y la región austral de Sudamérica. Buenos Aires: El Ateneo; 2002.

26.* Redford KH and Eisenberg JF. Mammals of the neotropics. The southern cone: Chile, Argentina, Uruguay, Paraguay. Chicago and London: The University of Chicago Press; 1992.

27.* Reid F. A field guide to the mammals of Central America and Southeast Mexico. New York: Oxford University Press; 1997.

28.* Reid F. A field guide to mammals of North America. Singapore: Houghton Mifflin; 2006.

29.* Tirira DG. Mamíferos de los bosques húmedos del noroccidente de Ecuador. Quito: Murciélago Blanco and PRIMENET; 2008.

30.* Wainwright MD. The mammals of Costa Rica. A natural history and field guide. Ithaca and Londres: Cornell University press; 2007.

31.* Whitaker JOJ. National Audubon Society field guide to mammals. New York: Alfred A. Knopf, Inc; 2007.

32.* Anderson RP and Handley CO. A new species of three-toed sloth (Mammalia: Xenarthra) from Panama, with a review of the genus Bradypus. Proc Biol Soc Wash. 2001;114: 1-33.

33.* Chiarello AG. Activity budgets and ranging patterns of the Atlantic forest maned sloth *Bradypus torquatus* (Xenarthra: Bradypodidae). J Zool. 1998;246: 1-10.

34.* Soibelzon E, Daniele G, Negrete J, Carlini AA and Plischuk S. Annual diet of the little hairy armadillo, *Chaetophractus vellerosus* (Mammalia, Dasypodidae), in Buenos Aires province, Argentina. J Mammal. 2007;88: 1319-1324.

35.* Vizcaíno SF and Milne N. Structure and function in armadillo limbs (Mammalia: Xenarthra: Dasypodidae). J Zool. 2002;257: 117-127.

36.* Vizcaíno SF, Fariña RA and Mazzetta GV. Ulnar dimensions and fossoriality in armadillos. Acta Theriol. 1999;44: 309-320.

37.* Escamilo BLL, Barrio J, Benavides F and Tirira DG. Northern Pudu, *Pudu mephistophiles* (De Winton, 1896). In: J. M. B. Duarte and S. González, editors. Neotropical Cervidology: biology and medicine of Latin American deer. Jaboticabal and Gland: FUNEP and IUCN; 2010. p. 133-139.

38.* Mysterud A, Pérez-Barbería FJ and Gordon IJ. The effect of season, sex and feeding style on home range area versus body mass scaling in temperate ruminants. Oecologia. 2001;127: 30-39.

39.* Pautasso AA. Mamíferos de la provincia de Santa Fe, Argentina. Buenos Aires: Ediciones Biológica; 2008.

40.* Perin MAA, Silva KFMd, Duarte JMB and Vogliotti A. Área de vida de cervos-do-pantanal (*Blastocerus dichotomus*) nascidos e reintroduzidos em una varzea do nordeste do estado de São Pablo (estaçao ecológica do Jataí), município de Luiz Antonio. Anais do VIII Congresso de Ecologia do Brasil: 1–2; 2007.

41.* Smith FA, Lyons SK, Morgan Ernest SK, Jones KE, Kaufman DM, et al. Body Mass of Late Quaternary Mammals. Ecology. 2003;84: 3403.

42.* Achaval F, Clara M and Olmos A. Mamíferos de la República Oriental del Uruguay. Montevideo: Facultad de Ciencias; 2004

43.* Audet AM, Robbins CB and Larivière S. *Alopex lagopus*. Mamm Species. 2002;713: 1-10.

44.* Balaguera-Reina SA, Cepeda A, Zárrate-Charry D and González-Maya JF. The state of knowledge of Western Mountain Coati *Nasuella olivacea* in Colombia, and extent of occurrence in the Northern Andes. Small Carniv Conserv. 2009;41: 35-40.

45.* Beisiegel B and Zuercher GL. *Speothos venaticus*. Mamm Species. 2005;783: 1-6.

46.* Castellanos A. Andean bear home ranges in the Intag region, Ecuador. Ursus. 2011;22: 65-73.

47.* Dalponte JC. *Lycalopex vetulus* (Carnivora: Canidae). Mamm Species. 2009;847: 1-7.

48.* Donadio E, Martino SD, Aubone M and Novaro AJ. Feeding ecology of the Andean hog-nosed skunk (*Conepatus chinga*) in areas under different land use in north-western Patagonia. J Arid Environ. 2004;56: 709-718.

49.* Hunter J and Caro T. Interspecific competition and predation in American carnivore families. Ethol Ecol Evol. 2008;20: 295-324.

50.* Kays RW. The behavior and ecology of olingos (*Bassaricyon gabbii*) and their competition with kinkajous (*Potus flavus*) in central Panama. Mammalia. 2000;64: 1-10.

51.* Larivière S. *Mustela vison*. Mamm Species. 1999;608: 1-9.

52.* Leite Pitman MRP and Williams RSR. Short-eared dog *Atelocynus microtis*. In: C. Sillero-Zubiri, M. Hoffmannand and D. W. Macdonald, editors. Canids: Foxes, Wolves, Jackals, and Dogs-Species Status and Conservation Action Plan. Gland and Cambridge: IUCN; 2004.

53.* Lucherini M and Luengos Vidal EM. *Lycalopex gymnocercus* (carnivora: canidae). Mamm Species. 2008;820: 1-9.

54.* Medellín RA, Ceballos G and Zarza H. *Spilogale pygmaea*. Mamm Species. 1998;600: 1-3.

55.* Napolitano C, Bennett M, Johnson WE, O'Brien SJ, Marquet PA, et al. Ecological and biogeographical inferences on two sympatric and enigmatic Andean cat species using genetic identification of faecal samples. Mol Ecol. 2008;17: 678-690.

56.* Novaro AJ. *Pseudalopex culpaeus*. Mamm Species. 1997;558: 1-8.

57.* Paisley S and Garshelis DL. Activity patterns and time budgets of Andean bears (*Tremarctos ornatus*) in the Apolobamba Range of Bolivia. J Zool. 2006;268: 25-34.

58.* Pasitschniak-Arts M and Larivière S. *Gulo gulo*. Mamm Species. 1995;499: 1-10.

59.* Pereira JA, Fracassi NG and Uhart MM. Numerical and spatial responses of Geoffroy's cat (*Oncifelis geoffroyi*) to prey decline in Argentina. J Mammal. 2006;87: 1132-1139.

60.* Rodríguez-Bolaños A, Sánchez P and Cadena A. Patterns of activity and home range of Mountain Coati *Nasuella olivacea*. Small Carniv Conserv. 2003;23: 1-5.

61.* Wrangham RW, Gittleman JL and Chapman CA. Constraints on group size in primates and carnivores: population density and day-range as assays of exploitation competition. Behav Ecol Sociobiol. 1993;32: 199-209.

62.* Ximenez A. *Felis geoffroyi*. Mamm Species. 1975;54: 1-4.

63.* Yensen E and Seymour KL. *Oreailurus jacobita*. Mamm Species. 2000;644: 1-6.

64.* Yensen E and Tarifa T. *Galictis cuja*. Mamm Species. 2003;728: 1-8.

65.* Yensen E and Tarifa T. *Galictis vittata*. Mamm Species. 2003;727: 1-8.

66.* Bonvicino CR, Oliveira JAD and D'Andrea PS. Guia dos roedores do Brasil, com chaves para gêneros baseadas em caracteres externos. Rio de Janeiro: Centro Pan-Americano de Febre Aftosa-OPAS/OMS; 2008.

67.* Campos C, Ojeda R, Monge S and Dacar M. Utilization of food resources by small and medium-sized mammals in the Monte Desert biome, Argentina. Austral Ecol. 2001;26: 142-149.

68.* Diaz GB, Ojeda RA and Rezende EL. Renal morphology, phylogenetic history and desert adaptation of South American hystricognath rodents. Funct Ecol. 2006;20: 609-620.

69.* dos Reis SF and Pessoa LM. *Thrichomys apereoides*. Mamm Species. 2004;741: 1-5.

70.* Ebensperger LA and Cofré H. On the evolution of group-living in the New World cursorial hystricognath rodents. Behav Ecol. 2001;12: 227-236.

71.* Ebensperger LA, Sobrero R, Campos V and Giannoni SM. Activity, range areas, and nesting patterns in the viscacha rat, *Octomys mimax*. J Arid Environ. 2008;72: 1174-1183.

72.* Gómez Villafañe IE, Miño M, Cavia R, Hodara K, Courtalón P, et al. Roedores. Guía de la provincia de Buenos Aires. Buenos Aires: L.O.L.A.; 2007.

73.* Justo ER, De Santis LJM and Kin MS. *Ctenomys talarum*. Mamm Species. 2003;730: 1-5.

74.* Lacey EA. Spatial and social systems of subterranean rodents. In: E. A. Lacey, J. L. Patton and G. N. Cameron, editors. Life underground: the biology of subterranean rodents Chicago: University of Chicago Press; 2000.

75.* Lacey EA and Sherman PW. The ecology of sociality in rodents. In: J. O. Wolff and P. W. Sherman, editors. Rodent societies: an ecological and evolutionary perspective. Chicago and Londres; 2007.

76.* Lessa EP, Vassallo AI, Verzi DH and Mora MS. Evolution of morphological adaptations for digging in living and extinct ctenomyid and octodontid rodents. Biol J Linn Soc. 2008;95: 267-283.

77.* Mares MA. Two new genera and species of halophytic desert mammals from isolated salt flats in Argentina. Occasional Papers, Museum of Texas Tech University. 2000;203: 1-27.

78.* Ojeda RA, Gonnet JM, Borghi CE, Giannoni SM, Campos CM, et al. Ecological observations of the red vizcacha rat, *Tympanoctomys barrerae*, in desert habitats of Argentina. Mastozool Neotrop. 1996;3: 183-191.

79.* Ojeda RA and Tabeni S. The mammals of the Monte Desert revisited. J Arid Environ. 2009;73: 173-181.

80.* Rosi MI, Cona MI, Roig VG, Massarini AI and Verzi DH. *Ctenomys mendocinus*. Mamm Species. 2005;777: 1-6.

81.* Samuels JX. Cranial morphology and dietary habits of rodents. Zool J Linn Soc. 2009;156: 864-888.

82.* Samuels JX and Van Valkenburgh B. Skeletal indicators of locomotor adaptations in living and extinct rodents. J Morphol. 2008;269: 1387-1411.

83.* Sobrero R, Campos VE, Giannoni SM and Ebensperger LA. *Octomys mimax* (Rodentia: Octodontidae). Mamm Species. 2010;42: 49-57.

84.* Spotorno AE, Zuleta CA, Valladares JP, Deane AL and Jiménez JE. *Chinchilla laniger*. Mamm Species. 2004;754: 1-9.

85.* Torres-Mura JC and Contreras LC. *Spalacopus cyanus*. Mamm Species. 1998;594: 1-5.

86.* Weisbecker V and Schmid S. Autopodial skeletal diversity in hystricognath rodents: functional and phylogenetic aspects. Z Saugetierkd. 2007;72: 27-44.

87.* DeLuycker AM. Notes on the yellow-tailed woolly monkey (*Oreonax flavicauda*) and its status in the protected forest of Alto Mayo, northern Peru. Primate Conserv. 2007;41-47.

88.* Di Fiore A and Campbell CJ. The atelines: variation in ecology, behavior, and social organization. In: C. J. Campbell, F. A., M. K.C., M. Panger and S. K. Beader, editors. Primates in perspective. New York: Oxford University Press; 2007. p. 155-185.

89.* Fernandez-Duque E. The Aotinae: social monogamy in the only nocturnal Haplorhines. In: C. J. Campbell, F. A., M. K.C., M. Panger and S. K. Beader, editors. Primates in perspective. New York: Oxford University Press; 2007. p. 139-154.

90.* Lindenfors P (2002) Sexually antagonistic selection on primate size. J Evol Biol. 2002;15: 595-607.

91.* Smith RJ and Jungers WL. Body mass in comparative primatology. J Hum Evol. 1997;32: 523-559.

92.* Townsend WR. *Callithrix pygmaea*. Mamm Species. 2001;665: 1-6.

93. Gower JC. A general coefficient of similarity and some of its properties. Biometrics. 1971;27: 857-871.

94. Laliberté E and Shipley B. FD: measuring functional diversity from multiple traits, and other tools for functional ecology. R package version 1.0-9; 2010.
