# Supplementary material for: Ecological Diversity in South American Mammals: Their Geographical Distribution Shows Variable Associations with Phylogenetic Diversity and Does Not Follow the Latitudinal Richness Gradient
Source: PLoS One. 2015 Jun 8;10(6):e0128264. doi: 10.1371/journal.pone.0128264 (PMC4460121; doi:10.1371/journal.pone.0128264)
Supplement: S3 File — (DOC) [file pone.0128264.s004.doc]

**S3 File.**

**A Fig.** **The geographical distribution of (a) highlands [1] and major rivers [2], (b) biomes [3] and (c) main climate types according to Köppen-Geiger classification [4]**. Abbreviations are: BF = Boreal forest/taiga, DXS = deserts and xeric shrublands, FGS = flooded grasslands and savannas, L= lake, M = mangroves, MFWS = Mediterranean forests, woodlands and scrub, MGS = montane grasslands and shrublands, RI = rock and ice, Te BMF = temperate broadleaf and mixed forests, Te CF = temperate conifer forests, Te GSS = temperate grasslands, savannas and shrublands, Tr-ST GSS = tropical and subtropical grasslands, savannas and shrublands, Tr-ST CF = tropical and subtropical coniferous forests, Tr-ST DBF = tropical and subtropical dry broadleaf forests, Tr-ST MBF = tropical and subtropical moist broadleaf forests, T = tundra. Maps are in Mollweide equal-area projection.

**References**

1. Hijmans RJ, Cameron SE, Parra JL, Jones PG, Jarvis A. Very high resolution interpolated climate surfaces for global land areas. Int J Climatol. 2005;25(15):1965-78.

2. ESRI. Global imagery and shaded relief included in ArcGIS 9.2 software. Redlands, CA2007.

3. Olson DM, Dinerstein E, Wikramanayake ED, Burgess ND, Powell GVN, Underwood EC, et al. Terrestrial ecoregions of the world: a new map of life on earth. BioScience. 2001;51(11):933-8.

4. Peel MC, Finlayson BL, McMahon TA. Updated world map of the Köppen-Geiger climate classification. Hydrology and earth system sciences discussions. 2007;4(2):439-73.
